# Circulating Extracellular Vesicles and Particles Derived From Adipocytes: The Potential Role in Spreading MicroRNAs Associated With Cellular Senescence

**DOI:** 10.3389/fragi.2022.867100

**Published:** 2022-08-09

**Authors:** Ionara Rodrigues Siqueira, Andressa de Souza Rodrigues, Marina Siqueira Flores, Eduarda Letícia Vieira Cunha, Madeleine Goldberg, Brennan Harmon, Rachael Batabyal, Robert J. Freishtat, Laura Reck Cechinel

**Affiliations:** ^1^ Programa de Pós-Graduação em Ciências Biológicas: Farmacologia e Terapêutica, Universidade Federal do Rio Grande do Sul, Porto Alegre, Brazil; ^2^ Programa de Pós-Graduação em Ciências Biológicas: Fisiologia, Universidade Federal do Rio Grande do Sul, Porto Alegre, Brazil; ^3^ Departamento de Farmacologia, Instituto de Ciências Básicas da Saúde, Universidade Federal do Rio Grande do Sul, Porto Alegre, Brazil; ^4^ Center for Genetic Medicine Research, Children’s National Hospital, Washington, WC, United States

**Keywords:** adipocyte-derived exosomes, microRNA, cellular senescence, aging, obesity, adipose tissue dysfunctions, tumors, cell cycle

## Abstract

Aging is associated with adipose tissue dysfunction and is recognized as a risk factor for shortened life span. Considering that *in vitro* findings have shown the involvement of microRNA in extracellular vesicles and particles (EVPs) on senescence, we hypothesized that circulating EVPs derived from adipocytes can be involved in the aging process *via* their microRNA cargo. We aimed to determine the microRNA profiles of circulating EVPs derived from adipocytes (FABP4+) from aged and young adult animals and to perform *in silico* prediction of their downstream signaling effects. Plasma was obtained from *Wistar* rats (3 and 21 months old), and adipocyte-derived EVPs were isolated using the commercially available kit. Fatty acid-binding protein 4 (FABP4) was used for adipocyte-derived EVPs isolation; microRNA isolation and microarray expression analysis were performed. The analysis revealed 728 miRNAs, 32 were differentially between groups (*p* < 0.05; fold change ≥ |1.1|), of which 15 miRNAs were upregulated and 17 were downregulated in circulating EVPs from aged animals compared to young adults. A conservative filter was applied, and 18 microRNAs had experimentally validated and highly conserved predicted mRNA targets, with a total of 2,228 mRNAs. Canonical pathways, disease and functions, and upstream regulator analyses were performed using IPA-QIAGEN, allowing a global and interconnected evaluation. IPA categories impacted negatively were cell cycle, cellular development, cellular growth and proliferation, and tissue development, while those impacted positively were “digestive system cancer” and “endocrine gland tumor.” Interestingly, the upregulated miR-15-5p targets several cyclins, such as CCND1 and CCND2, and miR-24-3p seems to target CDK4 (cyclin-dependent kinase 4); then potentially inhibiting their expression, both miRNAs can induce a negative regulation of cell cycle progression. In contrast, silencing of negative cell cycle checkpoint regulators, such as p21 and p16, can be predicted, which can induce impairment in response to genotoxic stressors. In addition, predicted targets, such as SMAD family members, seem to be involved in the positive control of digestive and endocrine tumors. Taken together, this exploratory study indicates that miRNA signature in circulating adipocyte-derived EVPs may be involved with the double-edged sword of cellular senescence, including irreversible proliferation arrest and tissue-dependent cancer, and seems to be suitable for further validation and confirmatory studies.

## Introduction

Aging is widely recognized as a risk factor for several diseases and is frequently associated with adipose tissue dysfunction characterized by changes in adipose tissue distribution, composition, and function (Niccoli and Partridge, 2012). Obesity is considered a risk factor for several diseases, through well-known underlying physiopathology by several international societies, including the American Medical Association (Poirier et al., 2006; Stoner and Cornwall, 2014; Saltiel and Olefsky, 2017). Obesity and overweight in adulthood are related to huge declines in life expectancy (Fontaine et al., 2003; Peeters et al., 2003); a vicious cycle between age-induced changes in adipose tissue dysfunction with accelerated aging, including age-related disease risk, has been identified (Jura and Kosak, 2016). In this context, visceral adipose tissue has been involved with age-induced systemic inflammation (Cevenini et al., 2010) because beyond a primary storage role, adipose tissue generates several bioactive compounds, such as cytokines, adipokines, hormones, and growth factors with autocrine, paracrine, and endocrine properties (Guilherme et al., 2008; Sun et al., 2011).

Among adipose tissue-derived circulating factors participating in inter-tissue and inter-organ cross-talk, the role of extracellular vesicles and particles (EVPs) has been raised (Ferrante et al., 2015; Castaño et al., 2018; Barcellos et al., 2020). Circulating microRNAs (miRNAs) can be carried by EVPs, such as exosomes and microvesicles, lipoproteins, and other ribonucleoprotein complexes. EVPs can protect miRNAs from endogenous RNases, in addition, internalization of these vesicles into cells is involved with cell communication, delivering miRNAs even to distant tissues. Recently, the role of circulating miRNAs transported by adipose tissue-derived EVPs has received much attention, since adipose tissue has a significant role as a resource of circulating exosomal miRNAs (Thomou et al., 2017).

*In vitro* and *in vivo* findings add to evidence showing the involvement of miRNA cargo in EVPs on metabolic process and the physiopathology of obesity. Castaño and colleagues (2018) demonstrated that obesity altered the miRNA profile, specifically levels of miR-27b-3p, miR-122, and miR-192, in plasma exosomes from mice. Several cell types, among them cardiomyocytes, preadipocytes, macrophages, pancreatic β, and hepatocytes, can be recipients of adipocytes-derived EVPs (Sano et al., 2014; Ferrante et al., 2015; Yu et al., 2018; Dang et al., 2019; Pan et al., 2019; Wen et al., 2020; Crewe et al., 2021; Gesmundo et al., 2021). For example, adipocyte-derived exosomes containing miR-27a were internalized by C2C12 skeletal muscle cells and *via* repression of PPARγ and its downstream genes seem to be related to insulin resistance induced by obesity (Yu et al., 2018). Hypertrophic adipocytes secrete exosomal miR-802-5p, which impaired HSP60 content and consequently the insulin signaling when incubated with neonatal rat ventricular myocytes (Wen et al., 2020). Moreover, these vesicles enriched with obesity-related miRNAs were injected into lean mice, inducing central obesity, hepatic steatosis, and glucose intolerance (Castaño et al., 2018).

Interestingly, EVPs have been considered central players in cellular senescence and, consequently, participating in the aging process (Xu and Tahara, 2013). The involvement of EVP miRNA released by senescent cells in the senescence of surrounding cells has been raised, moreover, EVPs seem to spread pro-senescence molecules to recipient cells (Weiner-Gorzel et al., 2015; Terlecki-Zaniewicz et al., 2018; Fulzele et al., 2019; Mensà et al., 2020). In this context, there is a growing body of evidence indicating the impact of aging on EVP profiles and its potential role as a mediator of intercellular communication, affecting physiopathological functions (Eitan et al., 2017; Bertoldi et al., 2018; Gomes de Andrade et al., 2018; Barcellos et al., 2020). Considering robust evidence about the involvement of obesity in aging cellular processes Carter et al., (2013) and Thomou et al. (2017) reported that adipose tissue has a significant role as a resource of circulating exosomal miRNAs, an intricated relationship between miRNA signature in circulating adipocytes-derived EVPs and the aging process can be raised.

We hypothesized that the aging-induced miRNAs changes into circulating adipocytes-derived EVPs (FABP4+) have a potential involvement with the aging process as a whole-body phenomenon and the vicious cycle between adipose tissue dysfunction in normal aging and age-related diseases. Our aim was to analyze the miRNA signature of circulating adipocyte derived EVPs from aged compared to young adult rats and perform *in silico* prediction of their downstream signaling effects, analyzing the predicted global effect on the physiological function of several miRNA changes, each one targeting numerous molecules in signaling pathways using bioinformatic tools.

## Materials and Methods

### Animals

Male *Wistar* rats of different ages, 3-month-old (*n* = 10) and 21-month-old (*n* = 11) were provided by Centro de Reprodução e Experimentação de Animais de Laboratório (CREAL) and maintained under standard conditions (12-h light/dark, 22°C ± 2°C) with food and water *ad libitum* (animals received commercial chow; Nuvilab Cr-1®, Nuvital Nutrientes S/A). Animals were not fasting before the decapitation, which took place between 9 and 10 a.m. The trunk blood was collected, after decapitation, in tubes with EDTA as an anticoagulant (4 ml) and centrifuged at 1,200 x g for 10 min to obtain plasma. The plasma was aliquoted (700 µl) and stored at −80°C until samples were used to isolate FABP4+ EVPs. Adiposity was significantly different between young adult and aged groups (body mass was 360 and 670 g; retroperitoneal fat mass was 6.7 and 43.9 g, respectively). The NIH “Guide for the Care and Use of Laboratory Animals” (NIH Publication No. 80-23, revised 1996) was followed in all experiments and the Local Ethics Committee (CEUA—Comissão de Ética no Uso de Animais—UFRGS; nr.29818) approved all animal procedures and experimental conditions.

### Adipocyte-Derived Extracellular Vesicles and Particles and MicroRNA Isolation

Fatty acid-binding protein 4 (FABP4) was used as a sensitive and specific marker for adipocyte-derived EVPs isolated from 700 ul of plasma of young adult and aged animals. Adipocyte-derived EVPs were isolated using the commercially available ExoQuick Precipitation Solution (System Biosciences, Mountain View, CA, United States). We then used an antibody complex and dextran-coated magnetic particles (StemCell Technologies, Vancouver, BC, Canada) to select FABP4+ EVPs in plasma. Adipocyte-derived EVPs were then prepared for RNA studies as described below.

Total RNA was extracted from adipocyte-derived EVPs using a commercially available kit (SeraMir Exosome RNA Amplification Kit; System Biosciences, Mountain View, CA, United States) according to manufacturer instructions; the average RNA concentration was 1001.98 ng/μl. RNA was labeled with Affymetrix® FlashTag™ Biotin HSR RNA Labeling Kit (Affymetrix, Santa Clara, CA, United States) according to standard procedures. Labeled RNA was hybridized to Affymetrix GeneChip microRNA 4.0 arrays and run using a Fluidics Station 450 protocol (FS450_002) (Affymetrix, Santa Clara, CA, United States). The resulting data were analyzed in Expression Console using RMA + DMBG (Affymetrix), then exported to Partek Genomics Suite (Partek, St. Louis, MO, United States) for the analyses.

IPA-QIAGEN indicated Canonical pathways, disease and functions, and upstream regulator analyses, categories evaluation allows to detect the global and interconnected effects of altered miRNAs and their respective targets. In addition, the upstream regulator analysis is a tool in IPA that can recognize potential upstream regulators of any gene or small molecule including miRNAs, transcription factors, cytokines, receptors, kinases, chemicals, and drugs.

### Statistical Analyses

Principal component analysis (PCA) and unsupervised hierarchical clustering (HC) were performed with Partek® Genomics Suite® software, v6.6. Analysis of variance (one-way ANOVA) was performed on Partek Genomics Suite to identify EVP miRNAs that were differentially expressed between aged and young-adult groups (*p* < 0.05 and fold changes ≥ |1.1|); those miRNAs were selected for further analysis. Differentially expressed miRNAs were analyzed with the use of QIAGEN IPA (QIAGEN Inc., https://digitalinsights.qiagen.com/IPA) to investigate the effects of the miRNAs on gene expression regulation during the aging process. The putative targets of miRNAs were determined using the IPA’s microRNA Target Filter, which identifies experimentally validated miRNA–mRNA interactions from TarBase, miRecords, and biomedical literature, as well as predicted miRNA–mRNA interactions from TargetScan. In this analysis we applied a conservative filter, selecting only experimentally validated and highly conserved predicted mRNA targets for each miRNA. Next, we used the mRNA target list in the core pathway analyses, which analyzed relationships among the mRNAs in our data set identifying canonical pathways, disease and functions, and upstream regulators. Canonical pathways with a *p*-value < 0.05 (Fischer’s exact test) were considered statistically significant, and the activation z-score was calculated to predict activation or inhibition of transcriptional regulators and disease and functions based on published findings accessible through Ingenuity Knowledge Base (Qiagen).

## Results

**FIGURE 1:**
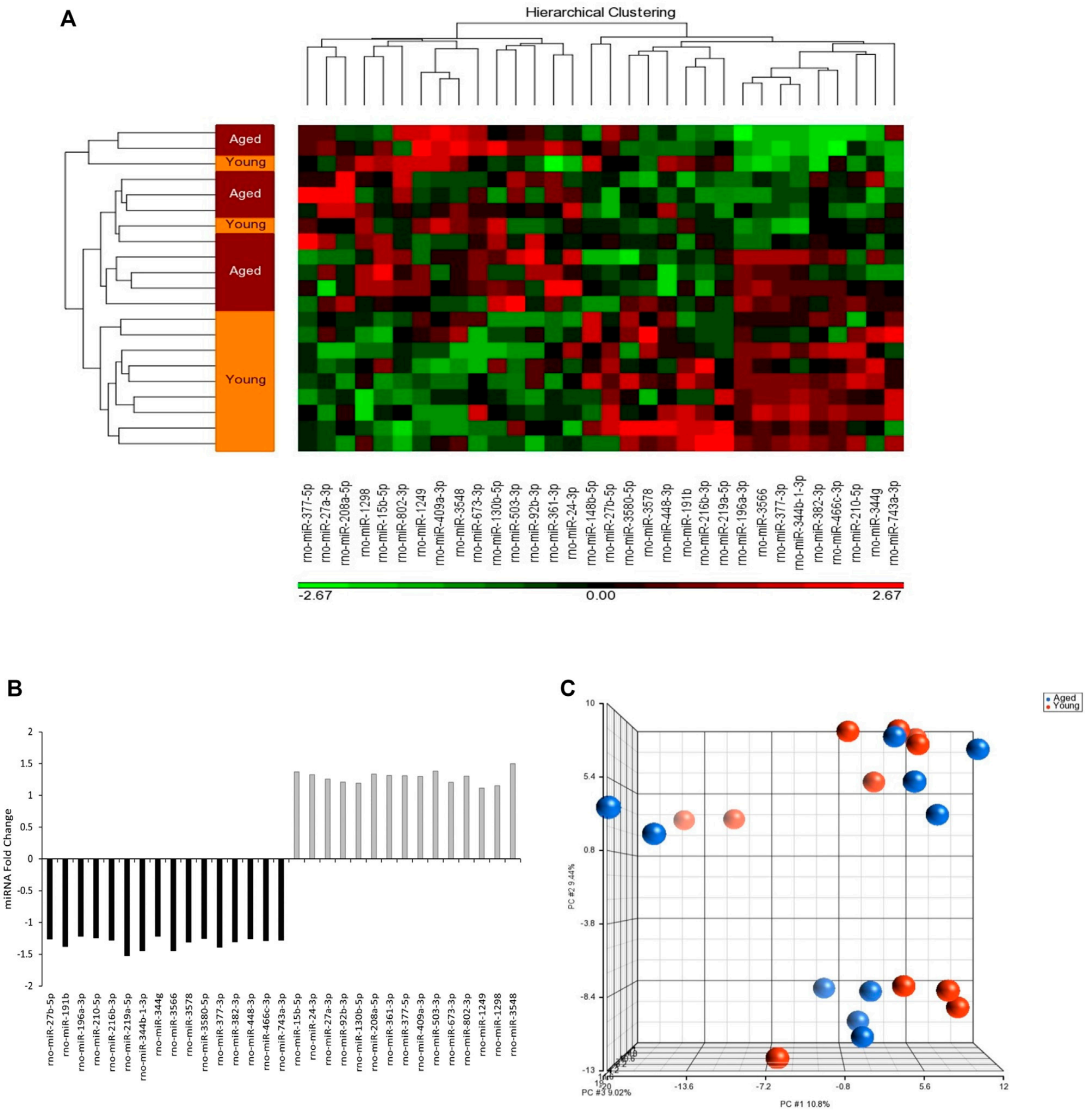
MicroRNAs (miRNAs) content in circulating adipocyte-derived EVPs obtained from aged Wistar rats differs from those of young adult ones. **(A)** Heatmap showing z-score and hierarchical clustering of the 32 miRNAs impacted in circulating adipocyte-derived EVPs by aging process. The microRNAs significantly expressed are shown on the bottom. Color gradation shows the relative microRNA content in circulating adipocyte-derived EVPs obtained from aged Wistar rats compared with those from young adult ones: green, downregulation; red, upregulation. **(B)** The fold change of miRNAs altered in circulating adipocyte-derived EVPs obtained from aged compared with young adult rats (One-way ANOVA, *p* < 0.05; fold change ≥ |1.1|). **(C)** Principal coordinates analysis (PCA) plot comparing differently miRNAs content in circulating adipocyte-derived EVPs from aged and young-adult animals. Each dot represents the overall miRNA expression in each animal. The distance between dots indicates their dissimilarity. PCA and hierarchical clustering were performed with Partek Genomics Suite (version 6.6; Partek, St. Louis, MO, United States).

The analysis of miRNA expression revealed 728 miRNAs in adipocyte-derived EVPs obtained from plasma. A heatmap of the hierarchical clustering shows different miRNA content in circulating adipocyte-derived EVPs between aged and young adult rats (Figure 1A). Of these miRNAs, 32 were differentially expressed between aged and young-adult groups (*p* < 0.05; fold change of 1.1), of which 15 miRNAs were significantly upregulated and 17 were downregulated in aged animals compared to young adults (Figure 1B). Global variability of adipocytes-derived EVPs miRNAs was explored with PCA analysis (Figure 1C), which demonstrated that the first three PCs are related to approximately 30% of the variability in data set (measured data) (PC1 10.8%, PC2 9.44%, and PC3 9.02%); the lack of clear distinct clustering in PCA scores plot indicated that the global variability (728 miRNAs in both ages) cannot be clustered in a smaller number of dimensions/components. Although PCA analyzes were unable to reduce the dimension of the dataset, as above cited, 32 were differentially expressed between aged and young-adult groups (Figure 1B).

The aging effect of circulating adipocyte-derived EVPs miRNA profiles on gene expression was investigated. We found that the altered miRNAs in circulating adipocyte-derived EVPs by aging putatively target 2,228 mRNAs (2,178 mapped and 50 unmapped targets).

**FIGURE 2:**
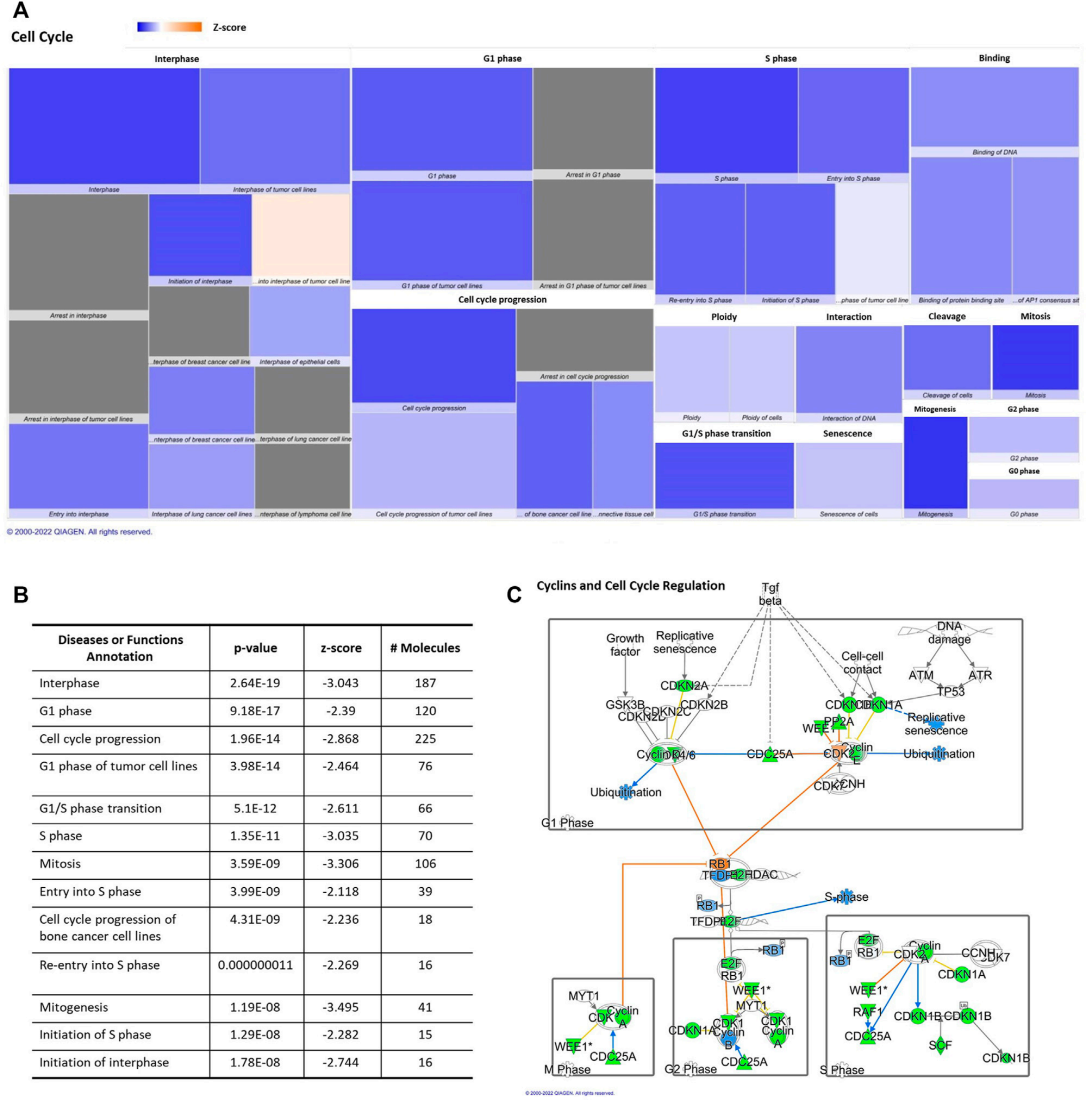
Aging-induced predicted effects of miRNAs content in circulating adipocyte-derived EVPs on Cell Cycle. **(A)** Heat map of the “Cell Cycle” among the top “molecular and cellular functions” categories. Each box represents one molecular and cellular function, its size represents gene enrichment. The heatmap is according to z-score values, where the color indicates the predicted increase or decrease status; higher z-scores would be represented by orange indicating activation, and lower z-scores was represented by blue indicating inhibition. **(B)** Annotations of the “Cell Cycle” category are impacted significantly by aging process, their z score, number of affected molecules, and *p* values. **(C)** mRNA Targets of microRNAs impacted by aging in circulating adipocyte-derived EVPs related with cell cycle canonical pathway. Green color indicates a predicted negative effect on mRNA targets, such as enzymes and transcription factors. miRNA-15b-5p targets cyclins (CCNs), CCND1, CCND2, CCND3, CCNE1, cyclin-dependent kinases (CDK) CDK6; miR-24-3p targets CCNA2, CDK1, and CDK4, while miR-92b-3p targets CCNE2. miR-15b-5p targets transcription factors, such as E2Fs isoforms, namely E2F3 and E2F7, while miR-24-3p targets E2F2. E2F is activated when retinoblastoma proteins (pRb) are phosphorylated and it dissociates from E2F (Table 1).

IPA displayed “Cell Cycle” among the top “molecular and cellular functions” categories impacted by differentially aging-regulated miRNAs. Downregulation of different regulatory molecules with a central role in cellular cycle and proliferation pathways, such as “cell cycle and cyclins” (Figure 2). This heat map is able to exhibit a huge effect on several phases of cell cycle (Figure 2A), the z score, number of affected molecules, and *p*-value were described in Figure 2B. All annotations of the “ cell cycle” category that were altered significantly had decreased predicted states, such as interphase, cell cycle progression, and mitosis (Figure 2B).

miRNAs altered by aging, miR-15b-5p, miR-24-3p, miR-92b-3p, miR-27a-3p, miR-377-5p, and miR-1249, in adipocytes-derived EVPs, target key classes of regulatory molecules of cell cycle progression, cyclins (CCNs) and cyclin-dependent kinases (CDK) enzymes, which can be downregulated in aged animals (Figure 2C, Table 1). The upregulated miRNAs target several cyclins and CDKs, miRNA-15b-5p targets CCND1, CCND2, CCND3, CCNE1, and CDK6; miR-24-3p targets CCNA2, CDK1, and CDK4, while miR-92b-3p targets CCNE2, then potentially inhibiting their expression, both altered miRNAs can be involved with negative regulation of cell cycle progression. In cell cycle, cyclin-CDK complexes induce phosphorylation of retinoblastoma proteins (pRb) and then the dissociation of E2F transcription factors from Rb family proteins, promoting activation of E2F proteins. In addition to targeting cyclin-CDK molecules, miR-15b-5p targets E2Fs isoforms, namely E2F3 and E2F7, while miR-24-3p targets E2F2. These transcription factors would modulate the expression of several DNA replications and cell cycle regulators genes; for example, E2F3 controls several genes, such as cyclin A and CDC6 (Cell Division Cycle 6), determining the rate of cellular proliferation by controlling the rate of DNA synthesis and G1/S transition (for review in Dimova and Dyson, (2005) (canonical pathway, Figure 2C, Table 1). On the other hand, negative regulators of cell cycle progression seem to be targeted by increased miRNAs in adipocyte-derived EVPs from aged animals (Table 1), such as miR-24-3p and miR-92b-3p target several cyclin-dependent kinase inhibitors, e.g., CDKN1A (p21), CDKN1B, and CDKN2A (p16), as well as regulators of the G2/M transition, specifically BRCA1 DNA repair associated (miR-24-3p), checkpoint kinase 1 (CHEK1 or chk1, miR-15b-5p), REPRIMO (miR-1249), and WEE1 (miR-15b-5p and miR-27a-3p). Interestingly, the “telomerase signaling” was one of the significant canonical pathways listed. miR-377-5p targets telomeric repeat binding factor 1 (TERF1) (Supplementary Figure S1) strongly involved with the structure of the telomere complex (Smith and Lange, 1997).

**TABLE 1:** Predicted targets involved with cell cycle regulation (and its expr fold change) of miRNAs in adipocyte-derived EVPs impacted by aging.

	Target	Predicted targets expression fold change	miRNA
BMI1	BMI1 proto-oncogene, polycomb ring finger	−1.37	rno-miR-15b-5p
BRCA1	BRCA1 DNA repair associated	−1.324	rno-miR-24-3p
BTRC	beta-transducin repeat containing E3 ubiquitin protein ligase	−1.37	rno-miR-15b-5p
CCNA2	Cyclin A2	−1.324	rno-miR-24-3p
CCND1	Cyclin D1	−1.37	rno-miR-15b-5p
CCND2	Cyclin D2	−1.37	rno-miR-15b-5p
CCND3	Cyclin D3	−1.37	rno-miR-15b-5p
CCNE1	Cyclin E1	−1.37	rno-miR-15b-5p
CCNE2	Cyclin E2	−1.208	rno-miR-92b-3p
CDC25A	Cell division cycle 25A	−1.37	rno-miR-15b-5p
CDK1	Cyclin-dependent kinase 1	−1.324	rno-miR-24-3p
CDK4	Cyclin-dependent kinase 4	−1.324	rno-miR-24-3p
CDK6	Cyclin-dependent kinase 6	−1.37	rno-miR-15b-5p
CDKN1A	Cyclin-dependent kinase inhibitor 1A (p21)	−1.208	rno-miR-92b-3p
CDKN1B	Cyclin-dependent kinase inhibitor 1B (p27)	−1.324	rno-miR-24-3p
CDKN2A	Cyclin-dependent kinase inhibitor 2A (p16)	−1.324	rno-miR-24-3p
CHEK1	Checkpoint kinase 1 (=chk1)	−1.37	rno-miR-15b-5p
E2F2	E2F transcription factor 2	−1.324	rno-miR-24-3p
E2F3	E2F transcription factor 3	−1.37	rno-miR-15b-5p
E2F7	E2F transcription factor 7	−1.37	rno-miR-15b-5p
FOXO1	Forkhead box O1	−1.256	rno-miR-27a-3p
KAT2B	Lysine acetyltransferase 2B	−1.208	rno-miR-92b-3p
MYC	MYC proto-oncogene, bHLH transcription factor	−1.324 (and −1.308)	rno-miR-24-3p (and rno-miR-377-5p)
PKMYT1	Protein kinase, membrane-associated tyrosinethreonine 1	−1.256	rno-miR-27a-3p
PLK1	Polo-like kinase 1	−1.37	rno-miR-15b-5p
PPM1D	Protein phosphatase, Mg2+Mn2+-dependent 1D	−1.37	rno-miR-15b-5p
PPP2R5C	Protein phosphatase 2 regulatory subunit B'gamma	−1.37	rno-miR-15b-5p
RAF1	Raf-1 proto-oncogene, serinethreonine kinase	−1.37	rno-miR-15b-5p
RPRM	Reprimo, TP53-dependent G2 arrest mediator homolog	−1.115	rno-miR-1249
SMAD3	SMAD family member 3	−1.324	rno-miR-24-3p
SMAD4	SMAD family member 4	−1.324	rno-miR-24-3p
WEE1	WEE1 G2 checkpoint kinase	−1.37 (and −1.256)	rno-miR-15b-5p (and rno-miR-27a-3p)
YWHAH	Tyrosine 3-monooxygenasetryptophan 5- monooxygenase activation protein eta	−1.37	rno-miR-15b-5p
YWHAQ	Tyrosine 3-monooxygenasetryptophan 5-monooxygenase activation protein theta	−1.256	rno-miR-27a-3p

**FIGURE 3:**
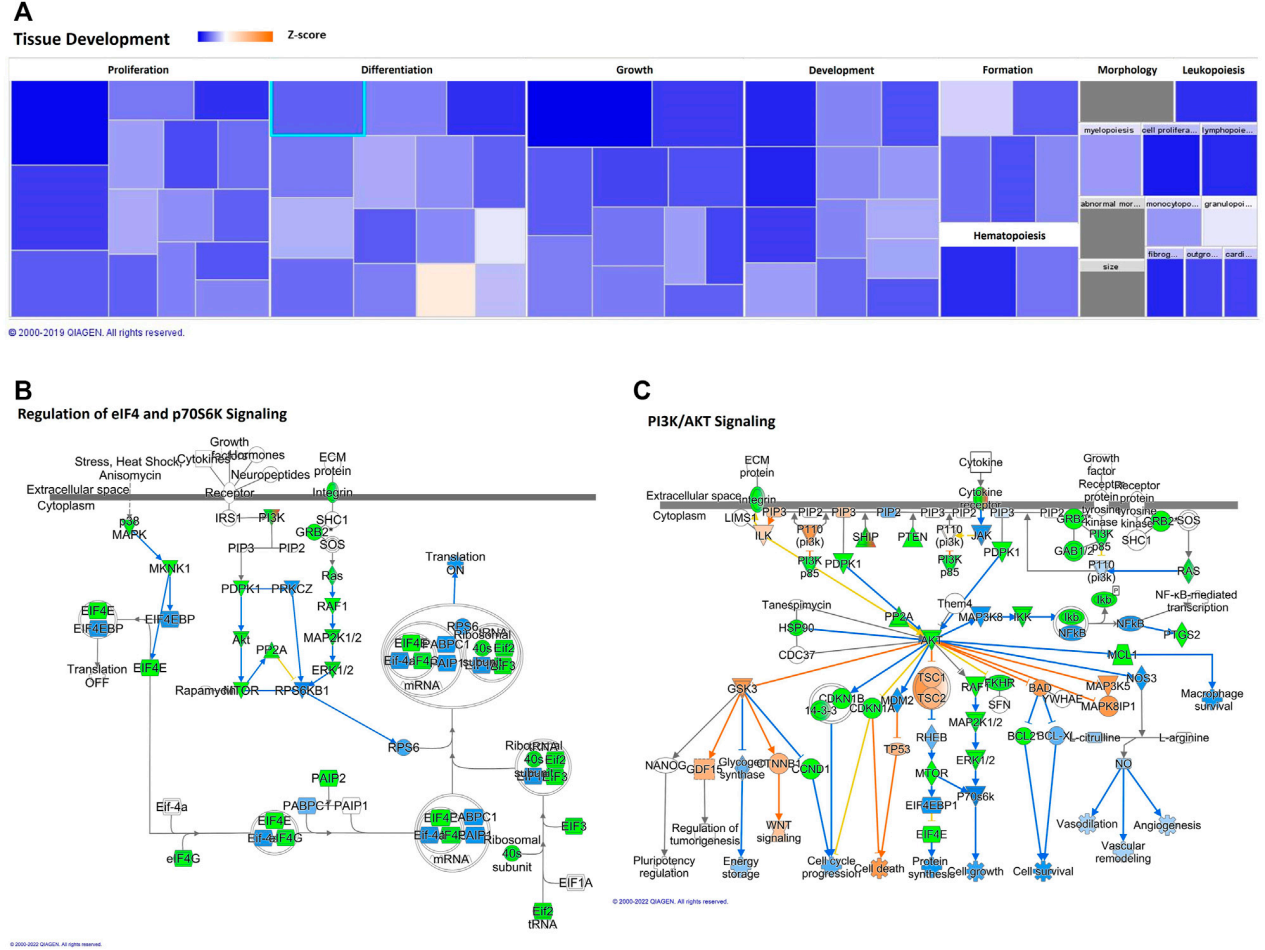
Aging-induced predicted effects of miRNAs content in circulating adipocyte-derived EVPs on Tissue Development. **(A)** Heat map of the “Tissue Development,” among the top “molecular and cellular functions” categories. Each box represents one molecular and cellular function, its size represents gene enrichment. The heatmap is according to z-score values, where the color indicates the predicted increase or decrease status; higher z-scores would be represented by orange indicating activation, and lower z-scores was represented by blue indicating inhibition. **(B)** Translational control canonical pathways, regulation of eukaryotic translation initiation factor 4 (eIF4) and p70S6K Signaling; mRNA Targets of microRNAs impacted by aging in circulating adipocyte-derived EVPs. Green color indicates predicted negative effect on mRNA targets, EIF3I (miR-24-3p), EIF4E (miR-15b-5p), EIF4G2 (miR-92b-3p), EIF4G2 (miR-210-5p and miR-377-5p), MAP2K1 and MAPK3 (miR-15b-5p). **(C)** Phosphoinositide-3-kinase (PI3K)/AKT Signaling Canonical Pathway impacting protein synthesis, proliferation, and survival. miR-92b-3p and miR-15b-5p target PIK3R3 and AKT3, respectively, and miR-503-3p targets the mammalian target of rapamycin (mTOR).

In addition, the “tissue development” and “cell growth and proliferation” category showed decreased predicted states, as well; the top two annotations of “tissue development” were “growth of connective tissue” and “proliferation of connective tissue cells,” as we can see in Table 2 and Figure 3. Besides cell cycle and pathways discussed below, such as cell death and apoptosis, protein turnover (protein synthesis and protein degradation) processes seem to be impacted by several diseases and function categories, such as “tissue development” and “cell growth and proliferation.” IPA revealed canonical pathways critical on regulation of the translation of messenger RNA (mRNA) for the top five molecular and cellular functions, which can be related to aging-induced reduced translational activity, such as p70S6K signaling (*p*-value = 0.00023; z = −2.837); EIF2 signaling (*p*-value = 0.0029; z = −3.273), regulation of eIF4 and p70S6K signaling (Figure 3B; *p*-value = 0.008; z = −2.673); Gα12/13 signaling (*p*-value = 0.0036; z = −2.065). Eukaryotic translation initiation factors (eIFs) are predicted targets of aging-related miRNAs in adipocytes-derived EVPs, EIF3I (miR-24-3p), EIF4E (miR-15b-5p), EIF4G2 (miR-92b-3p), and EIF4G2 (miR-210-5p and miR-377–5p). In addition, regulators of eIFs are targets of adipocytes-derived EVPs miRNAs impacted by aging, for example, mitogen-activated protein kinase (MAP2K1 and MAPK3, miR-15b-5p) and key molecules of PI3K/AKT/mTOR pathway (Figure 3).

**TABLE 2:** Disease or function annotations significantly altered in the “tissue development” category.

Disease or function annotation	*p* value	z-score	^#^ Molecules
Growth of connective tissue	2,15E-20	−5,87	171
Proliferation of connective tissue cells	2,67E-19	−4,983	157
Proliferation of epithelial cells	9,47E-16	−3,224	127
Growth of epithelial tissue	4,09E-15	−3,24	164
Differentiation of connective tissue cells	1,17E-14	−2,228	149
Growth of muscle tissue	5,37E-13	−2,381	97
Leukopoiesis	7,91E-13	−3,593	177
Development of mononuclear leukocytes	9,26E-13	−3,911	158
Hematopoiesis of mononuclear leukocytes	1,46E-12	−3,827	157
Proliferation of muscle cells	2,08E-12	−2,381	95
Differentiation of embryonic tissue	2,14E-12	−3,589	73
Differentiation of bone cells	5,5E-11	−2	91
Cell proliferation of fibroblasts	5,6E-11	−4,366	86
Growth of embryonic tissue	6,72E-11	−2,926	70
Growth of neurites	6,77E-11	−2,686	109
Lymphopoiesis	9,58E-11	−3,852	144
Development of connective tissue cells	2,08E-10	−2,713	67
Proliferation of neuronal cells	2,29E-10	−3,524	122
T cell development	1E-09	−4,01	117
Proliferation of mesenchymal cells	4,55E-09	−2,809	29
Development of epithelial tissue	5,54E-09	−3,217	114
Formation of gland	1,26E-08	−2,429	58
Differentiation of osteoblastic-lineage cells	1,5E-08	−2,296	60
Differentiation of osteoblasts	2,61E-08	−2,454	59
Formation of lymphoid tissue	3,48E-08	−2,004	76
Fibrogenesis	1,23E-07	−3,975	100
Proliferation of bone cells	1,23E-07	−2,281	27
Outgrowth of neurites	1,24E-07	−3,001	84
Growth of thymus gland	1,4E-07	−2,967	28
Proliferation of osteoblasts	1,41E-07	−2,288	24
Growth of bone tissue	1,88E-07	−2,286	27
Formation of muscle	1,91E-07	−2,692	82
Proliferation of thymocytes	3,11E-07	−2,554	26
Cartilage development	3,11E-07	−2,07	39
Cardiogenesis	4,55E-07	−3,082	107
Development of cardiovascular tissue	5,57E-07	−2,564	81

The potential effect of miRNAs profile in adipocytes-derived EVPs induced by aging on Phosphoinositide 3-kinase (PI3K)/AKT signaling that is related to protein synthesis, proliferation, and survival was predicted by IPA (*p*-value = 0.00000018, z = −2.88). The molecules belonging to this canonical pathway seem to be mostly under negative control by miRNAs altered by aging here described (Figure 3C). Phosphoinositide 3-kinase regulatory subunit 3 (PIK3R3) and AKT3 can be targeted by miR-92b-3p and miR-15b-5p, respectively, and miR-5030-3p targets the mammalian target of rapamycin (mTOR). However, the negative regulators can be regulated by protein phosphatase 2 (PP2A), because PPP2R5C (targeted by miR-15b-5p), and PTEN (as described below). AKT targets can be impacted by aging-induced changes of miRNAs into EVPs such as IKK (IκB kinase), a target of miR-15b-5p. Phosphatase and tensin homolog deleted on chromosome 10 (PTEN) signaling pathway was indicated by IPA as a canonical pathway affected potentially by aging-induced changes on miRNAs in adipocytes-derived EVPs (*p*-value = 0.00000006 z-score = +3.528; Supplementary Figure S2). It is relevant to note that although the z-score of this canonical pathway was +3.528 (a positive regulation), miR-92b-3p targets PTEN, which can induce decreases in PTEN levels. It is possible to infer the role of miRNAs on molecules which comprises complex regulation pathways, as different transcription factors pathways, such as c-JUN and nuclear factor-kappa B. miR-15b-5p target epidermal growth factor receptor (EGFR), a positive regulator of PTEN, the loss of this positive regulator could reduce PTEN levels. In addition, protein degradation systems can be impacted, such as “regulation of cellular mechanics by calpain protease” canonical pathway (*p*-value = 0.000007 z-score = −0.832). Even with z-score, our results indicate that protein degradation by calpain can be increased, since CAPN8 (calpain 8) is a predicted target of a downregulated miRNA, miR-210-5p (Supplementary Figure S3).

**FIGURE 4:**
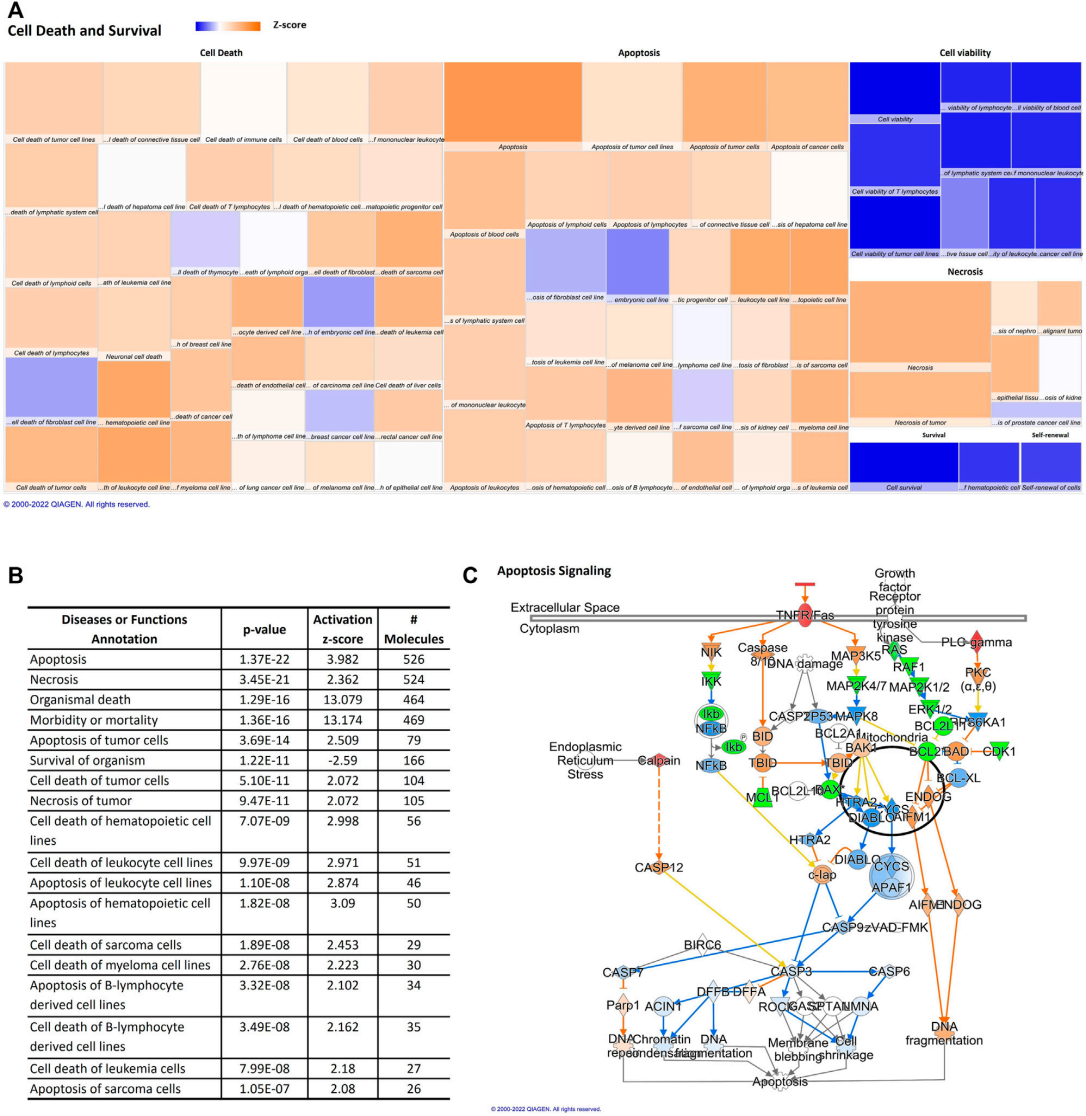
Aging-induced predicted effects of miRNAs content in circulating adipocyte-derived EVPs on Cell Death and Survival. **(A)** Heat map of the “Cell Death and Survival,” among the top “molecular and cellular functions” categories. Each box represents one molecular and cellular function, its size represents gene enrichment. The heatmap is according to z-score values, where the color indicates the predicted increase or decrease status; higher Z-scores were represented by orange indicating activation, and lower z-scores were represented by blue indicating inhibition. **(B)** Top Cell Death and Survival annotations; z score, number of affected molecules, and *p* values. **(C)** Apoptosis signaling canonical pathway. FAS/TNFR and CAPN8 are upregulated by miR-210-5p changes, miR-219a-5p promotes upregulation of FASLG, and miR-24-3p can be able to downregulate BCL-2, an anti-apoptotic molecule, bring evidence that extrinsic and intrinsic apoptotic pathways can be regulated by adipocyte-derived EVPs miRNAs in aging process.

Our data indicate that the miRNAs here described are related to the increase of pathological conditions and/or cell death during aging. An upregulation in cell death mechanisms and a downregulation in cell viability can be observed in the heat map (Figure 4A). Cell death and survival had annotations such as cell survival and cell viability that are predicted to be decreased, whilst apoptosis and necrosis are also annotations in cell death and survival category and are predicted to be increased. The z score, number of affected molecules, and *p*-value of top cell death and survival annotations were described in Figure 4B. Therefore, the aged miRNA profile promotes an increase in organismal death, morbidity, and mortality, which was observed in disease and function analysis. Moreover, cellular viabilities of different cell types seem to be decreased by changes on adipocytes-derived EVPs miRNAs profile during the aging process while cell death/apoptosis of hematopoietic cell lines, tumor cells, leukocyte cell lines, B-lymphocyte derived cell lines can be increased by adipocytes-derived EVPs miRNA profile from aged animals. Apoptosis signaling canonical pathway is upregulated by adipocyte-derived miRNAs (*p*-value = 0.000660693; Figure 4C). Adipocyte-derived EVPs miRNAs are predicted to exert regulation in both extrinsic and intrinsic apoptotic pathways. miR-210-5p is predicted to upregulate FAS/TNFR and CAPN8, whereas miR-219a-5p promotes upregulation of FASLG, a ligand of the extrinsic pathway. BCL-2, an important anti-apoptotic molecule in the intrinsic pathway, is downregulated by miR-24-3p.

**FIGURE 5:**
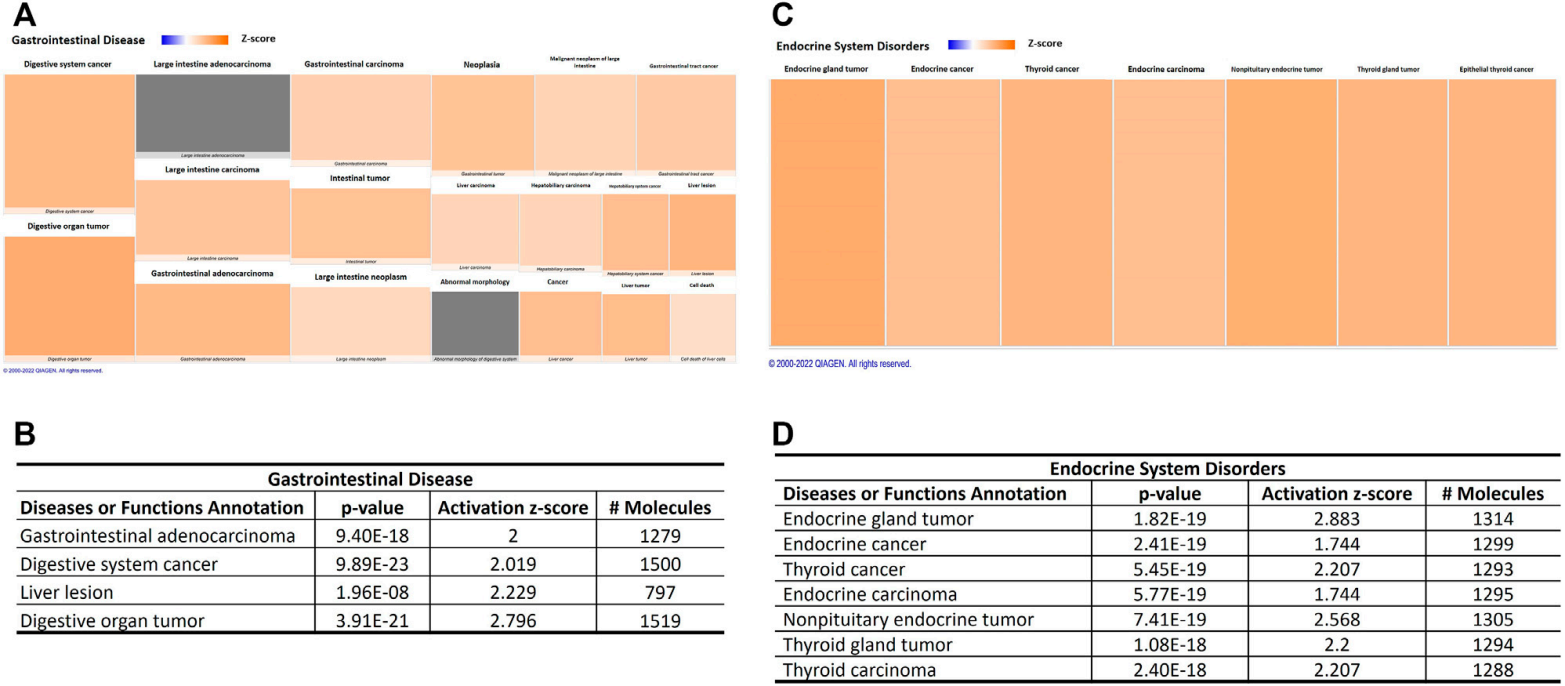
Aging-induced predicted effects of miRNAs content in circulating adipocyte-derived EVPs on endocrine and gastrointestinal tumors. **(A)** Heat map of the “gastrointestinal disease,” among the “ disease and functions” categories. Each box represents one molecular and cellular function, its size represents gene enrichment. The heatmap is according to z-score values, where the color indicates the predicted increase or decrease status; higher z-scores are represented by orange indicating activation, and lower z-scores would be represented by blue indicating inhibition. **(B)** Gastrointestinal disease annotations are impacted significantly by aging process; z score, number of affected molecules, and *p* values. **(C)** Heat map of the “ endocrine system disorders” annotation. **(D)** Endocrine system disorders annotations impacted significantly by aging-induced miRNAs changes in adipocytes-derived EVPs; z score, the number of affected molecules, and *p* values.

Interestingly, canonical pathway analysis revealed “molecular mechanisms of cancer” as the top signaling impacted by aging-induced changes in miRNA profile into circulating EVs derived from adipocytes (*p*-value = 0.0000000001; Table 3; Supplementary Table S1). This canonical pathway was listed as #NUM, indicating that its z-score algorithm cannot be predicted as increased or decreased (Shao et al., 2019). Diseases and functions indicate strongly the predicted effect of deregulated miRNAs in circulating EVPs on gastrointestinal and endocrine tumors, becausedigestive system cancer, digestive organ tumor, endocrine gland tumor, neck neoplasm, thyroid cancer, nonpituitary endocrine tumor, thyroid gland tumor, and thyroid carcinoma achieved z-score above 2 (Figure 5). However, canonical pathway analysis indicates a significant, but negative z-score, in several types of cancers, such as glioblastoma multiforme signaling (*p*-value = 0.0000000005; z score = −2.611) and glioma signaling (*p*-value = 0.00000001; z score = −3.13, Table 3). To try to figure out this apparent discrepancy, the predicted targets of the deregulated miRNAs by aging into circulating adipocytes-derived EVPs involved with canonical pathways and disease and functions were compared (Table 4). Table 4 shows relevant predicted targets of different canonical pathways of cancer, specifically molecular mechanisms of cancer (z = #NUM), those top with negative z-score: glioblastoma multiforme signaling, endocannabinoid cancer inhibition pathway, glioma signaling, pancreatic adenocarcinoma signaling, ovarian cancer signaling, comparing with those indicated by IPA for disease and functions with positive z-score, digestive system cancer, and digestive organ tumor. Interestingly, 17 predicted molecules targets, ARHGEF15, BAX, BCL2, BCL2L11, BMPR2, BRAF, BRCA1, CDC25A, CDK1, CDK17, CHEK1, GAB1, IHH, ITGA2, ITGA5, NOTCH1, RASA1, SMAD4, and SMAD7, appeared in molecular mechanisms of cancer and both digestive system cancer and endocrine gland tumor. Meanwhile, BBC3, LAMTOR3, and SMAD5 have been listed only in endocrine gland tumors and molecular mechanisms of cancer; digestive system cancer items had listed as JUN, PMAIP1, and SMAD9. It is remarkable that regulatory molecules of cell cycle described above, such as CCNs, CDKs, CDKIs, E2Fs, MAP2K1, MAP2K1, and MAPK3, were indicated as predicted targets in canonical pathways and disease and functions of cancer (Table 4).

**TABLE 3:** Top canonical pathways as predicted targets for aging-induced miRNAs content changes in circulating adipocyte-derived EVPs from aged animals compared to young adult (−log (p-value) ≥3; z-score ≥2 or ≤−2).

Ingenuity Canonical Pathways	−log(p-value)	z-score
Molecular Mechanisms of Cancer	10.1	#NÚM!
Glioblastoma Multiforme Signaling	9.5	−2.611
Glioma Signaling	8.14	−3.13
Cardiac Hypertrophy Signaling (Enhanced)	8	−5.658
Pancreatic Adenocarcinoma Signaling	7.63	−3.838
Estrogen-mediated S-phase Entry	7.34	−2.496
Ovarian Cancer Signaling	7.26	−3.153
PTEN Signaling	7.24	3.528
GADD45 Signaling	7.15	#NÚM!
PI3K/AKT Signaling	6.75	−2.887
Role of Osteoblasts. Osteoclasts and Chondrocytes in Rheumatoid Arthritis	6.61	#NÚM!

**TABLE 4:** Similar and different predicted targets of different top “canonical pathways” and “disease and functions” categories related to cancer.

miRNA impacted by aging	Target	FC/ targets	Molecular mechanisms of cancer	Glioblastoma multiforme signaling	Endocannabinoid cancer inhibition pathway	Glioma signaling	Digestive system cancer	Endocrine gland tumor	Type(s)
miR-15b-5p	AKT serine/threonine kinase 3	−1.37	AKT3	AKT3	AKT3	AKT3	AKT3	AKT3	Kinase
miR-24-3p	Rho guanine nucleotide exchange factor								
15	-1.324	ARHGEF15				ARHGEF15	ARHGEF1 5	Other	
miR-24-3p and miR-27a-3p	BCL2-associated X, apoptosis regulator	−1.324 and −1.256	BAX				BAX	BAX	Transporter
miR-24-3p and miR-27a-3p	BCL2 binding component 3	−1.324 and −1.256	BBC3					BBC3	Other
miR-15b-5p and miR-448-3p	BCL2 apoptosis regulator	−1.37 and 1.256	BCL2				BCL2	BCL2	Transporter
miR-24-3p and miR-92b-3p	BCL2-like 11	−1.324 and −1.208	BCL2L11				BCL2L11	BCL2L11	Other
miR-92b-3p	Bone morphogenetic protein receptor type 2	−1.208	BMPR2				BMPR2	BMPR2	Kinase
miR-503-3p	B-Raf proto-oncogene, serine/threonine kinase	−1.381	BRAF				BRAF	BRAF	Kinase
miR-24-3p	BRCA1 DNA repair associated	−1.324	BRCA1				BRCA1	BRCA1	Transcription regulator
miR-15b-5p	Cyclin D1	−1.37	CCND1	CCND1	CCND1	CCND1	CCND1	CCND1	Transcription regulator
miR-15b-5p	Cyclin E1	−1.37	CCNE1	CCNE1	CCNE1		CCNE1	CCNE1	Transcription regulator
miR-15b-5p	Cell division cycle 25A	−1.37	CDC25A				CDC25A		Phosphatase
miR-24-3p	Cyclin-dependent kinase 1	−1.324	CDK1				CDK1	CDK1	Kinase
miR-15b-5p	Cyclin-dependent kinase 17	−1.37	CDK17				CDK17	CDK17	Kinase
miR-24-3p	Cyclin-dependent kinase 4	−1.324	CDK4	CDK4		CDK4	CDK4	CDK4	Kinase
miR-15b-5p	Cyclin-dependent kinase 6	−1.37	CDK6	CDK6		CDK6	CDK6	CDK6	Kinase
miR-15b-5p	Cyclin-dependent kinase 8	−1.37	CDK8				CDK8	CDK8	Kinase
miR-92b-3p	Cyclin-dependent kinase inhibitor 1A	−1.208	CDKN1A	CDKN1A	CDKN1A	CDKN1A	CDKN1A	CDKN1A	Kinase
miR-24-3p	Cyclin-dependent kinase inhibitor 1B	−1.324	CDKN1B	CDKN1B	CDKN1B		CDKN1B	CDKN1B	Kinase
miR-24-3p	Cyclin-dependent kinase inhibitor 2A	−1.324	CDKN2A	CDKN2A		CDKN2A	CDKN2A	CDKN2A	Transcription regulator
miR-15b-5p	Checkpoint kinase 1	−1.37	CHEK1				CHEK1	CHEK1	Kinase
miR-24-3p	E2F transcription factor 2	−1.324	E2F2	E2F2		E2F2	E2F2	E2F2	Transcription regulator
miR-15b-5p	E2F transcription factor 3	−1.37	E2F3	E2F3		E2F3	E2F3	E2F3	Transcription regulator
miR-15b-5p	E2F transcription factor 7	−1.37	E2F7	E2F7		E2F7	E2F7	E2F7	Transcription regulator
miR-27a-3p	GRB2-associated binding protein 1	−1.256	GAB1				GAB1	GAB1	Other
miR-15b-3p and miR-27a-3p	Growth factor receptor bound protein 2	−1.37 and −1.324	GRB2	GRB2		GRB2	GRB2	GRB2	Other
miR-15b-5p	Indian hedgehog signaling molecule	−1.37	IHH				IHH	IHH	Enzyme
miR-15b-5p	Integrin subunit alpha 2	−1.37	ITGA2				ITGA2	ITGA2	Transmembrane receptor
miR-92b-3p	Integrin subunit alpha 5	−1.208	ITGA5				ITGA5	ITGA5	Transmembrane receptor
miR-15b-5p	Jun proto-oncogene, AP-1 transcription factor subunit	−1.37	JUN				JUN		Transcription regulator
miR-15b-5p	Late endosomal/lysoso mal adaptor, MAPK and MTOR activator 3	−1.37	LAMTOR3					LAMTOR3	Other
miR-219a-5p	Lymphoid enhancer binding factor 1	1.521	LEF1	LEF1	LEF1		LEF1	LEF1	Transcription regulator
miR-15b-5p	Mitogen-activated protein kinase 1	−1.37	MAP2K1	MAP2K1	MAP2K1	MAP2K1	MAP2K1	MAP2K1	Kinase
miR-15b-5p, miR-24-3p, miR-92b-3p and miR-27a-3p	Mitogen-activated protein kinase 4	−1.37, −1.324, −1.208 and −1.256	MAP2K4		MAP2K4		MAP2K4	MAP2K4	Kinase
miR-15b-5p	Mitogen-activated protein kinase 3	−1.37	MAPK3	MAPK3	MAPK3	MAPK3	MAPK3	MAPK3	Kinase
miR-24-3p and miR-377-5p	MYC proto-oncogene, bHLH	−1.324 and −1.308	MYC	MYC	MYC		MYC	MYC	Transcription regulator
	Transcription factor								
miR-24-3p and miR-27a-3p	Notch receptor 1	−1.324 and −1.256	NOTCH1				NOTCH1	NOTCH1	Transcription regulator
miR-219a-5p	Phosphatidylinosit ol-4-phosphate 3-kinase catalytic subunit type 2 gamma	1.521	PIK3C2G	PIK3C2G	PIK3C2G	PIK3C2G	PIK3C2G	PIK3C2G	Kinase
miR-92b-3p	Phosphoinositide-3-kinase regulatory subunit 3	−1.208	PIK3R3	PIK3R3	PIK3R3	PIK3R3	PIK3R3		Kinase
miR-24-3p, miR-377-5p and miR-448-3p	Phorbol-12-myristate-13-acetate-induced protein 1	−1.324, −1.308 and 1.256	PMAIP1				PMAIP1		Other
miR-15b-5p	Raf-1 proto-oncogene, serine/threonine kinase	−1.37	RAF1	RAF1	RAF1	RAF1	RAF1	RAF1	Kinase
miR-24-3p	RAP1A, member of RAS oncogenefamily	−1.324	RAP1A	RAP1A		RAP1A	RAP1A	RAP1A	Enzyme
miR-24-3p	RAP1B, member of RAS oncogenefamily	−1.324	RAP1B	RAP1B		RAP1B	RAP1B		Enzyme
miR-377-5p	RAS p21 protein activator 1	−1.308	RASA1				RASA1	RASA1	Transporter
miR-24-3p	RAS p21 protein activator 2	−1.324	RASD2	RASD2		RASD2	RASD2		Enzyme
miR-24-3p and miR-27a-3p	SMAD family member 4	−1.324 and −1.256	SMAD4				SMAD4	SMAD4	Transcription regulator
miR-24-3p and miR-27a-3p	SMAD family member 5	−1.324 and −1.256	SMAD5					SMAD5	Transcription regulator
miR-15b-5p	SMAD family member 7	−1.37	SMAD7				SMAD7	SMAD7	Transcription regulator
miR-27a-3p	SMAD family member 9	−1.256	SMAD9				SMAD9		Transcription regulator

Molecular mechanisms of cancer had z= ^#^num. Glioblastoma multiforme signaling, endocannabinoid cancer inhibition pathway, and glioma signaling, showed negative z-score, compared with those that IPA indicated positive z-score, digestive system cancer, and endocrine organ tumor.

IPA provides an analysis of upstream regulators, that can recognize potential upstream regulators of any gene or small molecule including miRNAs, transcription factors, cytokines, receptors, kinases, chemicals, and drugs. It is relevant to report that IPA identified 1,356 potential upstream regulators in our dataset (1,035 activated and 321 inhibited). Among those upstream regulators, we can find 14 miRNAs that are described here as differentially impacted by the aging process. miR-15b-5p (miR-16-5p is a synonym of miR-15b-5p) is the top upstream regulator, indicating its potential role in several biological processes. Indeed, IPA network analyses revealed an enormous number of potential targets in different cellular sites for miR-15b-5p (miR-16-5p; Supplementary Figure S4). The complete list of upstream regulators and their targets can be found in Supplementary Material Table S2.

## Discussion

Our work for the first time indicates that miRNA signatures of adipocytes-derived circulating EVPs from aged rodents can be related to the regulation of cell cycle progression, impacting proliferation arrest, and contribute to cancer, depending on the tissue, specifically gastrointestinal and endocrine tumors at distant sites, and provides additional support for the hypothesis that adipocytes-derived EVPs contribute substantially to called senescence-associated secretory phenotype in the aging process. Indeed, our results suggest the involvement of miRNA signature in adipocytes-derived EVPs in the vicious cycle of adipose tissue dysfunction and normal aging/age-related diseases (Jura and Kosak, 2016).

A growing body of evidence suggests that circulating adipocyte-derived EVPs can be a mechanism to spread molecules for all body systems, especially in the metabolic process and the physiopathology of obesity. Several cell types, among them cardiomyocytes, macrophages, pancreatic β, and hepatocytes, can be recipients of adipocytes-derived EVPs (Yu et al., 2018; Pan et al., 2019; Crewe et al., 2021; Dang and colleagues, 2019). The described upregulated miRNAs target several cyclins and CDKs, miRNA-15b-5p targets CCND1, CCND2, CCND3, CCNE1, CDK6; miR-24–3p targets CCNA2, CDK1, and CDK4, while miR-92b-3p targets CCNE2. Aging-induced miRNAs changes in adipocytes-derived EVPs can be involved with a negative regulation of cell cycle progression in recipient cells, besides, it is recognized that silencing of cyclins, such as CCNA2 and CCD1, can trigger cellular senescence (Laphanuwat and Jirawatnotai, 2019; Xu et al., 2019).

Interestingly, adipocyte-derived exosomes are internalized by C2C12 skeletal muscle cells and, *via* repression of PPARγ, seem to be related to insulin resistance induced by obesity (Yu et al., 2018). These authors also showed that the incubation of C2C12 cells with lipid-loaded adipocytes-derived exosomes increased FABP4 protein in C2C12 cells, additionally, FABP4 protein concentration was increased in skeletal muscle of obese mice (Yu et al., 2018), bringing evidence on the role of EVPs delivering their cargo of adipose tissue to skeletal muscle cells. In this context, it is possible to infer that miR-15b-5p, an upregulated miRNA in circulating adipocyte-derived EVPs here reported, can be involved with those findings of CCND1, given that this cyclin was strongly reduced in muscle stem cells from 20 to 22-months-old male mice, impairing myoblast proliferation and differentiation, including in response to regenerative conditions (Brett et al., 2020). In addition, Zhang et al. (2021) suggested the role of adipose tissue-derived EVPs cargo on bone remodeling events. Taken that tibia from 19-month-old aged female mice showed downregulated cyclins, CCNA2, CCND3, CCNE1-2, the upregulation of miRNA-15b-5p, miR-24-3p, and miR-92b-3p (and consequent silencing of cyclins) can be related, at least in part, to declines in bone mass and fracture in older adults, in the context of obesity. As well, downregulation of pro-proliferative transcription factors E2F1-4 was found in the bones of aged mice. Inhibition of E2F3 induced cell cycle arrest by inhibiting the induction of S phase (Leone et al., 1998). Moreover, bioinformatic analyses, functional clustering analysis, pathway enrichment, gene network interactions, and transcription factor of transcriptomic changes in tibia from old mice compared with young ones revealed “cell cycle” as a crucial category, including terms as organ development, cell cycle checkpoints and regulation of programmed cell death, chromatin assembly, DNA damage checkpoint, regulation of cell cycle, apoptosis, and regulation of metabolic process (Zhang et al., 2021).

On the other hand, miRNA profiling in circulating EVPs can reflect the tissue and cellular miRNA signatures. Meadows et al. (2020) reported changes in expression of visceral adipose tissue miRNAs from males and females Fischer344 × Brown-Norway hybrid rats at ages ranging 6, 15, 25, and 30-months-old, compared to 3-months-old. The heat map showed increases in miR-15b-5p, miR-24-3p, and miR-27a-3p levels in adipose tissue of male rats at older ages, while all aged groups compared to 3-month-old rats had increased levels of miR-24-3p and miR-27a-3p female rats. Bioinformatic, specifically functional enrichment analysis, showed that sexes had similar top categories impacted by miRNA signature in tissue adipose from 25-month-old rats compared with young adult ones. The top categories were cell death, hormone-mediated signaling pathway, cell cycle, cell proliferation, adipocyte differentiation, aging, cell differentiation, apoptosis, inflammation, and innate immunity. In this context, EVPs miRNA releasing machinery demands to raise the clearance of dysregulated miRNAs through EVs, given that miRNA sorting into EVs occurs selectively, can be related to findings of their target obtained in white adipose tissue by Li et al. (2021), because CCNA2 and CCNE1 were impacted in obese individuals, compared to lean individuals especially those with hyperinsulinemia.

Additionally, there is a prediction of silencing of CDC25A, a target of miR-15b-5p; then the loss of positive regulation by CDCA45 on cell cycle progression can be expected, once this enzyme would activate CDKs, such as CDK4 and CDK6. Taken together, it is possible to infer those aging-induced upregulated miRNAs in adipocytes-derived circulating EVPs, particularly miR-15b-5p and miR-24-3p, collaborate at least in part with cell cycle impairment, modulating key players in cell cycle progression and DNA replication, limiting including the tissue ability to repair the damage.

Several upregulated miRNAs in plasma adipocyte-derived EVPs from aged rats target critical component cell cycle checkpoints, which can be related to the failure of these checkpoints leading to aberrant cell cycle progression, impaired mitosis with an accumulation of DNA damage, and increased genomic instability during aging. Interestingly, the upregulated miRNAs here described, miR-92b-3p targets CDKN1A (p21); and miR-24-3p targets CDKN2A (p16) and CDKN1B (p27). Jackson et al. (2002) reported the synergic effect of genetic alterations, loss of p21 and p27, and exposure to the chemical carcinogen, urethane, because urethane-treated p21-null mice had accelerated tumor onset and increased tumor formation; while tumor growth rate, especially salivary tumors, once tumors appeared, was increased in p27-null mice. Furthermore, our data, shows that a negative modulation of CDK inhibitors, can be involved with those findings of *in vivo* distribution patterns of senescence markers since a single-cell transcriptomics study reported that only 2%–3% of cells in organs of old mice are p16- positive cells (Tabula Muris, 2020); on the other hand, DNA double-strand breaks and lipid peroxidation can achieve up to 70% of cardiomyocytes (Ogrodnik, 2021).

Previous studies on senescent cells-derived EVPs have indicated an upregulation of CDK inhibitors, p16, p27, and p53 as mechanisms of cellular senescence, especially in human fibroblasts, which could inhibit the proliferation of abnormal cells and consequently might induce tumor suppression (Tanaka and Takahashi, 2021). However, to the best of our knowledge, our work is the first showing a potential *ex vivo* evidence of the involvement of miRNAs signature in circulating adipocytes-derived EVPs on silencing of both cell cycle checkpoint genes, p21, p27, and p16, which could induce an impairment on response to genotoxic stressors, such as DNA damage, and then abrogates cell cycle arrest.

In this context, a cell cycle arrest might eventually be overpassed even with reduced levels of cell cycle positive regulatory molecules, such as cyclins and CDKs (Kozar and Sicinski, 2005). In accordance, Takeuchi et al. (2010) described that mice with knockout of p16 INK4a, and p21 WAF1/CIP1 genes had significantly shortened life spans related to tumor diseases. It is remarkable that p21-deficient mice develop early spontaneous tumors (approximately 4 months), including hematopoietic, endothelial, and epithelial tumors (Martin-Caballero et al., 2001).

Regulators of the G2/M transition are also targeted by aging-induced miRNAs changes in adipocytes-derived EVPs. miR-24-3p targets BRCA1 ( breast cancer susceptibility gene 1) that is involved with DNA repair and transcriptional regulation in response to DNA damage, also it is essential to the maintenance of chromosomal stability, thereby protecting the genome from damage. BRCA1 phosphorylation is essential for G2/M checkpoints in the DNA damage response since it activates CHK1 kinase and acts with p53, p21, and p27 (Yoshida and Miki, 2004). Wee1, another negative regulator of entry in mitosis (G2 to M transition) that inactivates cyclin B1-CDK1 complex, seems to be downregulated by adipocyte-derived EVPs (specifically, by miR-15b-5p and miRNA27a-3p). Potapova et al. (2011) described that reduced CDC25 concomitantly with Wee1 and Myt1 kinases inhibition induced a mitotic “collapse” status; as described above CDC25a and Wee1 are targets of miR-15b-5p, predicted silencing of Wee1 and CDC25A can contribute to aberrant mitosis in the aging process. In the same way, the predicted silencing of checkpoint kinase 1 (CHEK1, chk1), a target of miR-15b-5p, can induce a proliferation impairment by a defective G2/M DNA damage checkpoint (Liu et al., 2000). It is relevant to note that a Polo-like kinase (PLK), specifically PLK1, is also a target of miR-15b-5p. These kinases have a central role in mitosis *per se* since they regulate centrosome maturation, spindle assembly, and cytokinesis (Barr et al., 2004). miR-15b-5p also targets a component of SCF complex, specifically BTRC (beta transducin repeat containing E3 ubiquitin-protein ligase), related to the regulation of the cell cycle with periodic proteolysis of many regulators of the cell cycle by the ubiquitin-proteasome system, among several of them, such as cyclins, CDKs, E2Fs, CKIs (p21), and Wee1, it is possible to cite additional substrates, such as IκB, CREB, and β-catenin, an important mediator of Wnt signaling, degraded as a result of BTRC-dependent ubiquitylation (Thompson et al., 2021). Silencing of BTRC by miR-15-5p can be involved at least in part with decreased proteasome activity widely described during the aging process in various model systems and has been associated with aging and age-associated diseases.

miRNA cargo in adipocytes-derived EVPs modified by the aging process did not impact directly p53, however, our findings indicate a negative regulation on determinant steps of cell cycle control (negative checkpoints), for example, p16INK4A, p21WAF1/CIP1, p27Kip1, CHK1, Cdk1. It is relevant to comment that CHK1 is a downstream kinase that propagates the signal of damage, acting on p53. This loss of negative control by aging-induced changes on adipocytes-derived EVPs miRNA cargo can be involved with deregulation of cell cycle, allowing proliferation even with DNA damage. In accordance, accumulated evidence indicates irregular cell division, such as aneuploidy, as a hallmark of cancer cells, and the role of checkpoints in cancer development. Several steps seem to be impacted by miRNA changes described above, including checkpoint-control proteins which would trigger cell cycle arrest in response to chromosomal defects or DNA damage, among several others, BRCA1, CHEK1, p16, and p14. In this context, the loss or severe reduction of p27 (here target by is miR24-3p) is widely observed in human tumors, impairing p27-induced block cell division.

It is impossible to affirm at this moment who are the potential recipient cells of circulating adipocytes-derived EVPs impacted by aging, however, we could infer that miRNAs into adipocytes-derived circulating EVPs impacted by aging have a potential role in the age-related decline life span of bone marrow stromal cells (Stenderup et al., 2003), since mesenchymal stem cells have reduced CCNE1 and CCND2 expression (Wilson et al., 2010); in addition, these cells seem to abrogate cell cycle checkpoints with loss of p21 (50-fold) and Rb1 expression, as well CHEK2 (Wilson et al., 2010). Loss of control of a series of timed and interrelated events can result in mitotic catastrophe which can drive or at least collaborate with overall tissue degeneration during aging.

In this context, it is possible to infer that adipocytes-derived EVPs secreting pro-tumorigenic miRNAs inhibiting cell cycle checkpoints that drive can be involved with the relationship between obesity and the aging process on the increased risk of developing several types of cancer by driving dormant tumor cells to awaken and form a proliferative lesion. Furthermore, the senescence-associated secretory phenotype of aging-induced changes in adipocytes-derived EVPs can have pro-tumorigenic functions; a dual effect on tumorigenesis has been recognized depending on cellular origin, an inducer of senescence, such as oncogene and/or DNA damage due to a varied senescence-associated secretory phenotype secretome (for review, Faget et al., 2019).

This scenario can be accompanied by an impairment of telomerase signaling, since miR-377-5p targets telomeric repeat binding factor 1 (TERF1) that interacts with and is involved with the looping of duplex telomeric DNA, beginning the structure of the telomere complex, and can inhibit telomerase (Smith and Lange, 1997). The silencing of TERF1 can lead to genomic instability, chromosome end fusion, and failure in regulating telomerase recruitment and telomere maintenance. It is relevant to point out, that some studies reported an association between short telomeres and early death (Jaskelioff et al., 2011; Mather et al., 2011; Gruber et al., 2021). Furthermore, shortened telomeres or telomerase mutations were observed in people with diseases characterized by premature aging (Fani et al., 2020). It was previously reported that fibroblasts from telomeric repeat binding factor 1 (TERF1)-deficient mice show rapid induction of senescence with increased cancer and degenerative phenotypes (Martínez et al., 2009).

It is remarkable to point out that the top canonical pathway was “molecular mechanisms of cancer” without a predicted z-score algorithm, what can be involved with the fact of diseases and functions revealed positive impacts on gastrointestinal and endocrine tumors, meanwhile canonical pathway analysis showed negative z-score in types of cancers, such as glioblastoma multiforme signaling and glioma signaling. This negative control by aging-deregulated miRNAs into circulating adipocytes-derived EVPs on some tumor cells seems to be related to inhibiting cellular proliferation, in agreement with the cellular senescence concept that is considered as a mechanism for tumor suppression that would inhibit the proliferation of abnormal cells, acting on regulatory molecules of cell cycle, such as cyclins, E2Fs, and several kinases, CDKs and MAP kinases, above discussed. Our results concerning miRNA cargo in adipocytes-derived EVPs agree with those about the involvement of EVPs cargo from several types and origins associated with cellular senescence (Fulzele et al., 2019; Mensà et al., 2020; Tanaka and Takahashi, 2021).

PTEN regulates central cellular functions such as tumor suppressor, genomic stability, cellular proliferation, survival, and motility, acting on several proteins and lipid substrates. PTEN can be modulated by aging-induced changes on miRNAs in adipocytes-derived EVPs, specifically an upregulated miRNA, miR-92b-3p. PTEN is listed in most diseases and functions annotations. Considering the controversial aspects between canonical pathway z-score (predicted positive regulation) and direct effect on PTEN by miR-92b-3p (predicted negative regulation), it is possible to infer the role of miRNAs on molecules that comprise its exceptionally complex regulation pathways, as different transcription factors pathways, such as c-JUN and nuclear factor-kappa B, and miRNAs at the post-transcriptional level, such as mir-221, what can have consequently a tumor promoter role (He et al., 2021). miR-15b-5p targets the epidermal growth factor receptor (EGFR), a positive regulator of PTEN, the loss of this positive regulator could reduce PTEN levels. However, this may be an oversimplification, the silencing of PTEN by miR-92b-3p (direct effect) and miR-15b-5p (indirect effect *via* EGFR) may contribute to tumor susceptibility, since reduced PTEN levels have been observed in several tumors, such as colorectal cancer, hepatocellular carcinoma, and thyroid cancers (Correia et al., 2014; Bermúdez Brito et al., 2015). Kotelevets et al. (2020) advocate that increases in PTEN expression can induce a response of tumor cells to therapies, restoring or increasing its tumor suppressor activities. In this context, modulation of miRNAs content into EVPs can be an approach to alleviate obesity as a risk factor in malignancies.

PI3K/AKT signaling, under negative regulation by miRNAs of circulating EVPs from aged animals, would regulate several key cellular functions, such as cell survival and proliferation, angiogenesis and protein synthesis, transcription, and apoptosis. The molecules belonging to this canonical pathway seem to be mostly under negative control by miRNAs altered by aging here described. PI3K converts phosphatidylinositol bisphosphate (PIP2) lipids to phosphatidylinositol trisphosphate (PIP3). miR-92b-3p and miR-15b-5p, respectively, target PIK3R3 and AKT3; negative regulators of PI3K/AKT pathway, the protein phosphatase 2 and PTEN can be regulated by miRNAs here described. Interestingly, the predicted target of miR-15b-5p, AKT3, would regulate different pathways, including those linked to cell cycle, survival, and growth, e.g., controlling cellular proliferation by modulating several targets, such as cyclins and CDKs; AKT3 silencing can be involved and can potentiate the direct effect on these specific targets.

In addition, pathways with positive control showed specific targets, such as the SMAD family, as well as in biological function “cell cycle G1/S checkpoint.” This family composes the signaling effector of activated TGF-β receptors, which activates SMAD2 and SMAD3 to generate a complex with SMAD4; this complex translocates to the nucleus and regulates the transcription of target genes. The inactivation of components of TGF-β signaling pathways, as the potential silencing of SMADs by mRNA-24-3p here described, can contribute to cell proliferation, metastasis, and cancer development. SMAD4 mutation with reduced active protein has been related to most pancreatic carcinomas (for review by Ullah et al., 2018). Another potential target is BMI1 (B cell-specific Moloney murine leukemia virus integration site 1), a member of the polycomb family of epigenetic repressors. Hu et al. (2019) reported that aged mice had reduced Bmi1 expression in bone marrow stromal cells, these authors demonstrated the involvement of BMI1 with increased marrow adipocytes in aging. It is possible that miRNA-15b-5p in EVPs and the consequent silencing of BMI1 in surrounding adipocytes can contribute to this scenario.

The aging process has been widely related to loss of cellular proteostasis, an impairment of the balance between protein synthesis and degradation systems (Santra et al., 2019). Relevant canonical pathway for regulation of the translation of messenger RNA (mRNA) seems to be impacted by miRNAs content into circulating EVPs here described, such as p70S6K signaling; eIF2 signaling, regulation of eIF4, and p70S6K signaling, which can be related to the aging-induced reduced translational activity. In this context, eukaryotic translation initiation factors (eIFs) are predicted targets of aging-related miRNAs in adipocytes-derived EVPs, specifically EIF3I, EIF4E, EIF4G2, and EIF4G2. Besides, eIFs levels and activities are regulated by several signaling pathways (Hao et al., 2020), whose components seem to be a target of miRNAs here described, such as mitogen-activated protein kinase (MAP2K1 and MAPK3, both miR-15b-5p) phosphatidylinositol 3-kinase (PI3K)/AKT, AKT3 miR-15b-5p), and mammalian target of rapamycin (mTOR) pathways (mTOR, miR-503-3p). In addition, p70S6K is required for cell growth and cell cycle progression. Our results suggest that miRNAs profile in circulating adipocyte-derived EVPs obtained from aged Wistar rats collaborate at least in part with translation initiation impairment, which can impact cellular growth and development. Although to our knowledge the involvement of adipocytes-derived EVPs has not been evaluated, the reduced levels of IGF-1, AKT, mTOR, and P70 S6 kinase (p70S6K) levels were found in the muscles of aged rats (Yeo et al., 2020). Moreover, our results indicate that protein degradation by CAPN8 (calpain 8), which is found especially in surface mucus in the stomach cells and in the goblet cells of the intestines (Sorimachi et al., 1993; Hata et al., 2010), can be increased, because this is a predicted target of a downregulated miRNA, miR-210-5p. Altogether, these target molecules that belong to pathways have been implicated with sarcopenia, muscle quantity, or quality loss, in aged animals, consequently, adipocytes-derived EVPs can be involved with sarcopenic obesity, concomitant muscle loss, and fat accumulation in the aging process. Sarcopenia as an age-related disease has been associated with complications or comorbidities, such as frailty, falls, fractures, and cachexia.

Corroborating what was previously discussed, loss of cellular checkpoint responses that can lead to a cell cycle deregulation, for example, it is not surprising that apoptosis signaling is upregulated by adipocyte-derived miRNAs. Interestingly, adipocyte-derived miRNAs target the intrinsic and extrinsic apoptotic pathway through downregulation of the anti-apoptotic protein BCL-2 and upregulation of death receptors. The binding of ligands (FASLG) to death receptors (FAS and TNFR) provides a rapid and efficient route to apoptosis, initiating the caspase cascade (Kumar et al., 2005). FAS/TNFR and FASLG have predicted targets of miR-210-5p and miR-219a-5p, respectively. Interestingly, miR-219a-5p was previously associated with the regulation of neuronal apoptosis and death (Yan et al., 2019). Moreover, calpains are key candidate targets involved directly with the apoptosis pathway, they act as pro-apoptotic proteases by activating caspases (Smith & Schnellmann, 2012). Since miRNA in adipocyte-derived EVs, specifically miR-210-5p, target CAPN8 (calpain 8), this interaction may be highly relevant in aging. The adipocyte-derived EVs miRNAs exert a complex regulation, balancing anti- and pro-apoptotic signals in the apoptosis pathway, in line with this, miR-24-3p exerts a negative regulation on BCL-2. BCL-2 is an apoptosis inhibitory protein that plays a vital role in controlling the life span of cells. Corroborating our data, repression of BCL-2 was associated with increased stress-induced apoptosis in senescent human diploid fibroblasts by increasing the sensitivity of senescent fibroblasts to apoptosis induction (Ryu et al., 2007).

Interestingly, we also observed a predicted increase in apoptosis of leukocytes, lymphocytes, and hematopoietic cell lines. Studies indicate that in elderly individuals, lymphocyte apoptosis may be correlated with immunosenescence as well as increased autoimmune disorders (Pollack and Leeuwenburgh, 2001; Xu et al., 2020). In our data, we observed that IKKβ (inhibitor of nuclear factor-kappa B kinase subunit beta) is a common molecule present in all different cell lines apoptosis. In line with this, it was already shown that altered expression of IKKβ resulted in increased TNF-α-induced apoptosis in lymphocytes in aged humans. In addition, it is worth mentioning that Behrens et al. (2011) showed a difference in the necrosis-to-apoptosis ratio in aged and young human T lymphocytes. In agreement with our data, they also observed higher levels of apoptosis with increasing age, demonstrating an increased susceptibility of human T lymphocytes to apoptosis induced by the FAS ligand.

It is interesting to note that there is a consensus about age-promoting properties of exposures to some compounds, also called “gerontogenic,” such as tobacco. Cigarette smoking has been involved with premature aging, accelerating aging, and, consequently, is a risk of age-related diseases and a link between cigarette smoking and cellular senescence has been widely raised (Poussin et al., 2021). In accordance with our findings and hypothesis, Shi et al. (2016) reported that miR-16-5p (a synonym of miR-15b-5p) had increased levels in plasma from healthy smokers, since miR-15b-5p (miR-16-5p is a synonym of miR-15b-5p) was the top one upstream regulator, indicating its potential role on several biological processes, including cell cycle deregulation.

Beyond the role of miRNA profile in circulating EVPs induced by aging as central mediators of intercellular communication, this study can bring new insights and might open new avenues for further studies, such as new therapies focused on miRNAs derived from adipose tissue. Besides, it can support the need for treatments to induce weight loss, such as educational campaigns stimulating healthy eating and/or exercise programs and/or bariatric surgery in the elderly population. In this context, specific changes in miRNA content in circulating adipocytes-derived EVPs might be used as biomarkers of efficacy. Interestingly, Hubal et al. (2017) evaluated the miRNA profile of adipocyte-derived EVPs from African-American female subjects with obesity (age: 38.5 ± 6.8 years), before and 1 year after bariatric surgery. These authors demonstrated that among surgery-impacted miRNAs, it is miRNA-15b-5p (whose symbol can be miR-16-5p and other miRNAs w/seed AGCAGCA; FC–1.59), indicating a reduction by bariatric surgery-induced weight loss (BMI −18.6 ± 5.1 kg/m^2^; *p* < 0.001).

Altogether, the potential involvement of increased adiposity and the consequent adipocytes-derived EVPs signature on cellular senescence was here reported, their miRNAs as inducers of and/or contributors to organismal damage. To the best of our knowledge, this is the first report evaluating the aging-induced miRNA changes in circulating adipocyte-derived EVPs (FABP4+) with *in silico* prediction of their downstream signaling effects. Our data obtained from an exploratory approach raised evidence about the role of adipocyte-EVPs miRNAs signatures from aged rats on susceptibility to cell damage, depending on the tissue, which predisposes to cell death or aberrant mitoses contributing to cancer development, and the need for future confirmatory studies to confirm these exploratory results. Indeed, adipocytes-derived circulating EVPs can be involved in the vicious circle between aging and obesity, propagating senescence signals.

## Data Availability

The original contributions presented in the study are included in the article/Supplementary Materials; further inquiries can be directed to the corresponding author.
