# Supplementary material for: Circulating Extracellular Vesicles and Particles Derived From Adipocytes: The Potential Role in Spreading MicroRNAs Associated With Cellular Senescence
Source: Front Aging. 2022 Aug 9;3:867100. doi: 10.3389/fragi.2022.867100 (PMC9395989; doi:10.3389/fragi.2022.867100)
Supplement: Supplementary file 1 [file Table1.docx]

**Supplementary Material Table S1.** canonical pathways as predicted targets for aging-induced miRNAs content changes in circulating adipocyte-derived EVPs from aged animals compared to young adult (-log (p-value) ≥3; z-score ≥2 or ≤-2).

| **Ingenuity Canonical Pathways** | **-log(p-value)** | **z-score** |
| --- | --- | --- |
| Molecular Mechanisms of Cancer | 10.1 | #NÚM! |
| Glioblastoma Multiforme Signaling | 9.5 | -2.611 |
| Glioma Signaling | 8.14 | -3.13 |
| Cardiac Hypertrophy Signaling (Enhanced) | 8 | -5.658 |
| Pancreatic Adenocarcinoma Signaling | 7.63 | -3.838 |
| Estrogen-mediated S-phase Entry | 7.34 | -2.496 |
| Ovarian Cancer Signaling | 7.26 | -3.153 |
| PTEN Signaling | 7.24 | 3.528 |
| GADD45 Signaling | 7.15 | #NÚM! |
| PI3K/AKT Signaling | 6.75 | -2.887 |
| Role of Osteoblasts. Osteoclasts and Chondrocytes in Rheumatoid Arthritis | 6.61 | #NÚM! |
| Osteoarthritis Pathway | 6.58 | -3.124 |
| Neuregulin Signaling | 6.47 | -3.13 |
| Aryl Hydrocarbon Receptor Signaling | 6.44 | -2.785 |
| Prostate Cancer Signaling | 6.35 | #NÚM! |
| Acute Myeloid Leukemia Signaling | 5.95 | -3.153 |
| Myc Mediated Apoptosis Signaling | 5.89 | #NÚM! |
| Role of Macrophages. Fibroblasts and Endothelial Cells in Rheumatoid Arthritis | 5.59 | #NÚM! |
| Role of Tissue Factor in Cancer | 5.58 | #NÚM! |
| Regulation of the Epithelial-Mesenchymal Transition Pathway | 5.58 | #NÚM! |
| STAT3 Pathway | 5.51 | -4.082 |
| TGF-β Signaling | 5.34 | -2.982 |
| Mouse Embryonic Stem Cell Pluripotency | 5.31 | -2.558 |
| Chronic Myeloid Leukemia Signaling | 5.31 | #NÚM! |
| Bladder Cancer Signaling | 5.25 | -3.051 |
| PPAR Signaling | 5.24 | 3.128 |
| Systemic Lupus Erythematosus In B Cell Signaling Pathway | 5.07 | -3.244 |
| Human Embryonic Stem Cell Pluripotency | 5.04 | #NÚM! |
| Melanoma Signaling | 4.93 | -2.309 |
| Corticotropin Releasing Hormone Signaling | 4.88 | -3 |
| IL-8 Signaling | 4.86 | -5.145 |
| Role of NANOG in Mammalian Embryonic Stem Cell Pluripotency | 4.67 | -2.496 |
| Colorectal Cancer Metastasis Signaling | 4.6 | -4.333 |
| Adrenomedullin signaling pathway | 4.57 | -4.004 |
| IL-6 Signaling | 4.29 | -3.545 |
| NF-κB Signaling | 4.28 | -3.413 |
| HGF Signaling | 4.25 | -2.837 |
| Ephrin Receptor Signaling | 4.24 | -4.315 |
| IGF-1 Signaling | 4.23 | -3.153 |
| Salvage Pathways of Pyrimidine Ribonucleotides | 4.22 | -4.146 |
| G-Protein Coupled Receptor Signaling | 4.22 | #NÚM! |
| HMGB1 Signaling | 4.2 | -2.524 |
| Thyroid Cancer Signaling | 4.17 | #NÚM! |
| Protein Ubiquitination Pathway | 4.15 | #NÚM! |
| PDGF Signaling | 4 | -2.357 |
| Endocannabinoid Developing Neuron Pathway | 4 | -2.4 |
| Non-Small Cell Lung Cancer Signaling | 3.94 | -2.887 |
| ErbB Signaling | 3.94 | -2.982 |
| Insulin Receptor Signaling | 3.93 | -2.858 |
| Endometrial Cancer Signaling | 3.91 | -2.887 |
| FAK Signaling | 3.87 | #NÚM! |
| 14-3-3-mediated Signaling | 3.74 | -2.236 |
| PAK Signaling | 3.74 | -2.828 |
| P2Y Purigenic Receptor Signaling Pathway | 3.74 | -2.4 |
| Altered T Cell and B Cell Signaling in Rheumatoid Arthritis | 3.73 | #NÚM! |
| FLT3 Signaling in Hematopoietic Progenitor Cells | 3.72 | -3.638 |
| Neurotrophin/TRK Signaling | 3.71 | -3.357 |
| FGF Signaling | 3.65 | -3.3 |
| p70S6K Signaling | 3.64 | -2.837 |
| BMP signaling pathway | 3.58 | -3.153 |
| UVC-Induced MAPK Signaling | 3.56 | -3.464 |
| UVB-Induced MAPK Signaling | 3.47 | -3.051 |
| Prolactin Signaling | 3.37 | -2.324 |
| Cholecystokinin/Gastrin-mediated Signaling | 3.35 | -4.146 |
| Regulation of IL-2 Expression in Activated and Anergic T Lymphocytes | 3.32 | #NÚM! |
| Clathrin-mediated Endocytosis Signaling | 3.31 | #NÚM! |
| GDNF Family Ligand-Receptor Interactions | 3.22 | -2.138 |
| EGF Signaling | 3.22 | -2.496 |
| Erythropoietin Signaling | 3.22 | #NÚM! |
| G Beta Gamma Signaling | 3.19 | -3.273 |
| HER-2 Signaling in Breast Cancer | 3.17 | #NÚM! |
| Synaptogenesis Signaling Pathway | 3.14 | -5.154 |
| VEGF Signaling | 3.12 | -3.153 |
| NRF2-mediated Oxidative Stress Response | 3.1 | -3.357 |
| ILK Signaling | 3.06 | -3.402 |
| Melanocyte Development and Pigmentation Signaling | 3.03 | -3.153 |
| Hepatic Cholestasis | 3 | #NÚM! |
| Adipogenesis pathway | 3 | #NÚM! |
