# Supplementary material for: Circulating Extracellular Vesicles and Particles Derived From Adipocytes: The Potential Role in Spreading MicroRNAs Associated With Cellular Senescence
Source: Front Aging. 2022 Aug 9;3:867100. doi: 10.3389/fragi.2022.867100 (PMC9395989; doi:10.3389/fragi.2022.867100)
Supplement: Supplementary file 2 [file Table2.docx]

**Supplementary Material Table S2.** Top upstream regulators identified by IPA analysis of miRNAs content in circulating adipocyte-derived EVPs obtained from aged and young adult Wistar rats.

| **Upstream Regulator** | **Predicted Activation State** | **z-score** | **p-value of overlap** |
| --- | --- | --- | --- |
| miR-16-5p (and other miRNAs w/seed AGCAGCA) | Activated | 22.352 | <2.41E-256 |
| miR-24-3p (and other miRNAs w/seed GGCUCAG) | Activated | 15.545 | 2.41E-256 |
| miR-27a-3p (and other miRNAs w/seed UCACAGU) | Activated | 14.842 | 8.08E-241 |
| miR-92a-3p (and other miRNAs w/seed AUUGCAC) | Activated | 14.195 | 1.05E-208 |
| miR-377-5p (and other miRNAs w/seed GAGGUUG) | Activated | 10.721 | 4.73E-122 |
| miR-130b-5p (and other miRNAs w/seed CUCUUUC) | Activated | 9.713 | 2.43E-108 |
| miR-503-3p (and other miRNAs w/seed GAGUAUU) | Activated | 8.888 | 1.86E-83 |
| miR-4524a-5p (and other miRNAs w/seed UAGCAGC) | Activated | 9.501 | 1.81E-78 |
| miR-448-3p (and other miRNAs w/seed UGCAUAU) | Inhibited | -6.882 | 6.8E-77 |
| miR-219a-5p (and other miRNAs w/seed GAUUGUC) | Inhibited | -8.807 | 2.57E-76 |
| miR-27b-5p (miRNAs w/seed GAGCUUA) | Inhibited | -6.863 | 3.64E-76 |
| miR-208a-5p (and other miRNAs w/seed AGCUUUU) | Activated | 8.426 | 3.33E-73 |
| miR-1249-3p (and other miRNAs w/seed CGCCCUU) | Activated | 8.185 | 8.28E-71 |
| miR-210-5p (and other miRNAs w/seed GCCACUG) | Inhibited | -6.306 | 3.19E-67 |
| miR-802-3p (and other miRNAs w/seed CGGAGAG) | Activated | 7.878 | 1.77E-66 |
| miR-148a-5p (and other miRNAs w/seed AAGUUCU) | Inhibited | -5.939 | 5.78E-59 |
| miR-382-3p (miRNAs w/seed AUCAUUC) | Inhibited | -5.98 | 1.41E-43 |
| miR-503-5p (miRNAs w/seed AGCAGCG) | Activated | 7.329 | 8.04E-37 |
| miR-103-3p (and other miRNAs w/seed GCAGCAU) | Activated | 8.093 | 1.21E-30 |
| miR-128-3p (and other miRNAs w/seed CACAGUG) | Activated | 5.861 | 6.74E-24 |
