# Supplementary material for: Circulating Extracellular Vesicles and Particles Derived From Adipocytes: The Potential Role in Spreading MicroRNAs Associated With Cellular Senescence
Source: Front Aging. 2022 Aug 9;3:867100. doi: 10.3389/fragi.2022.867100 (PMC9395989; doi:10.3389/fragi.2022.867100)

**Supplementary Figures.**

**S1 Figure 1.** Telomerase Signaling


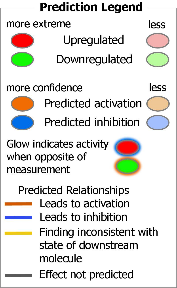

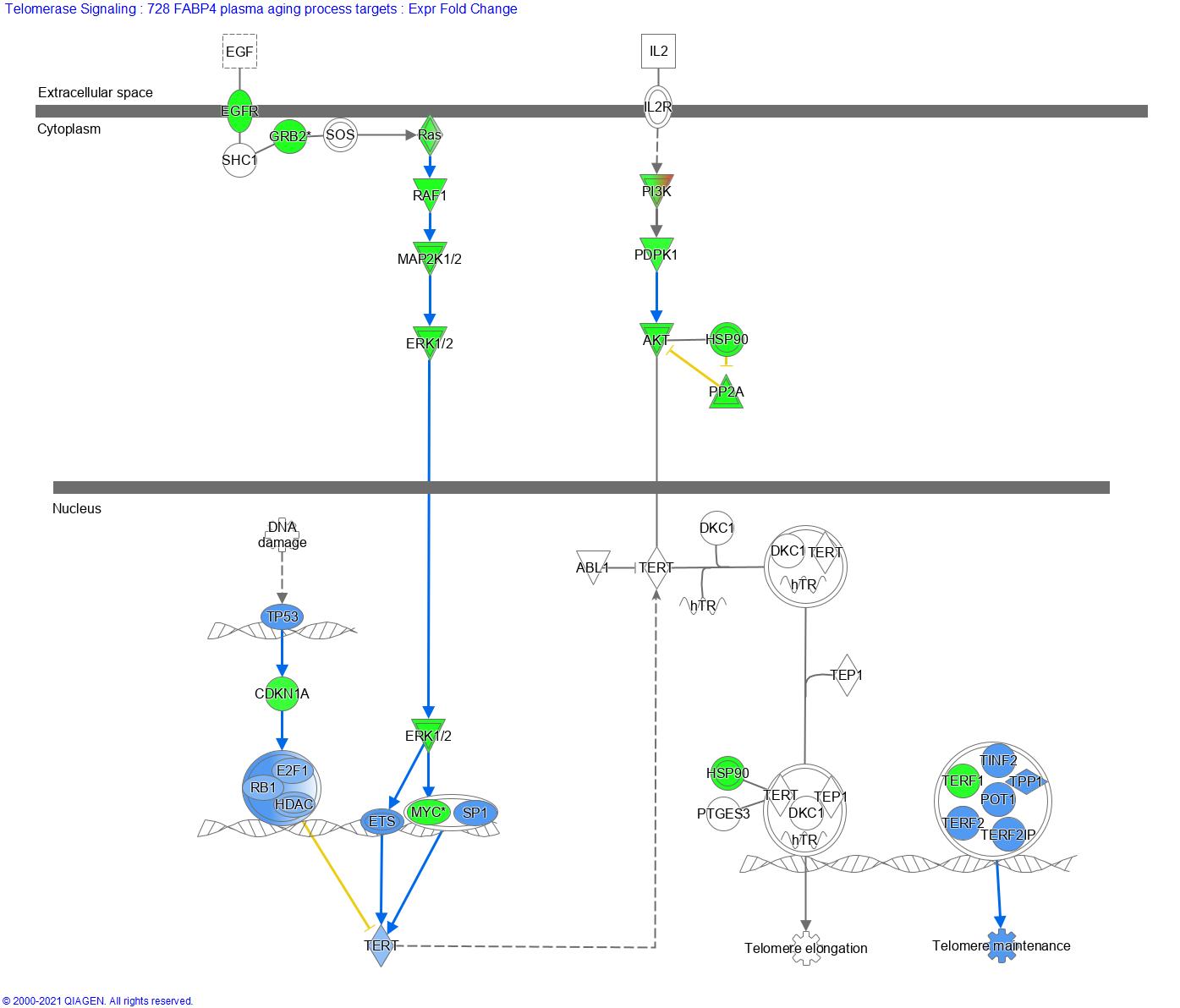


**S2 Figure 2.** PTEN Signaling


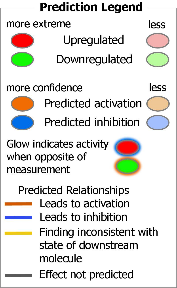

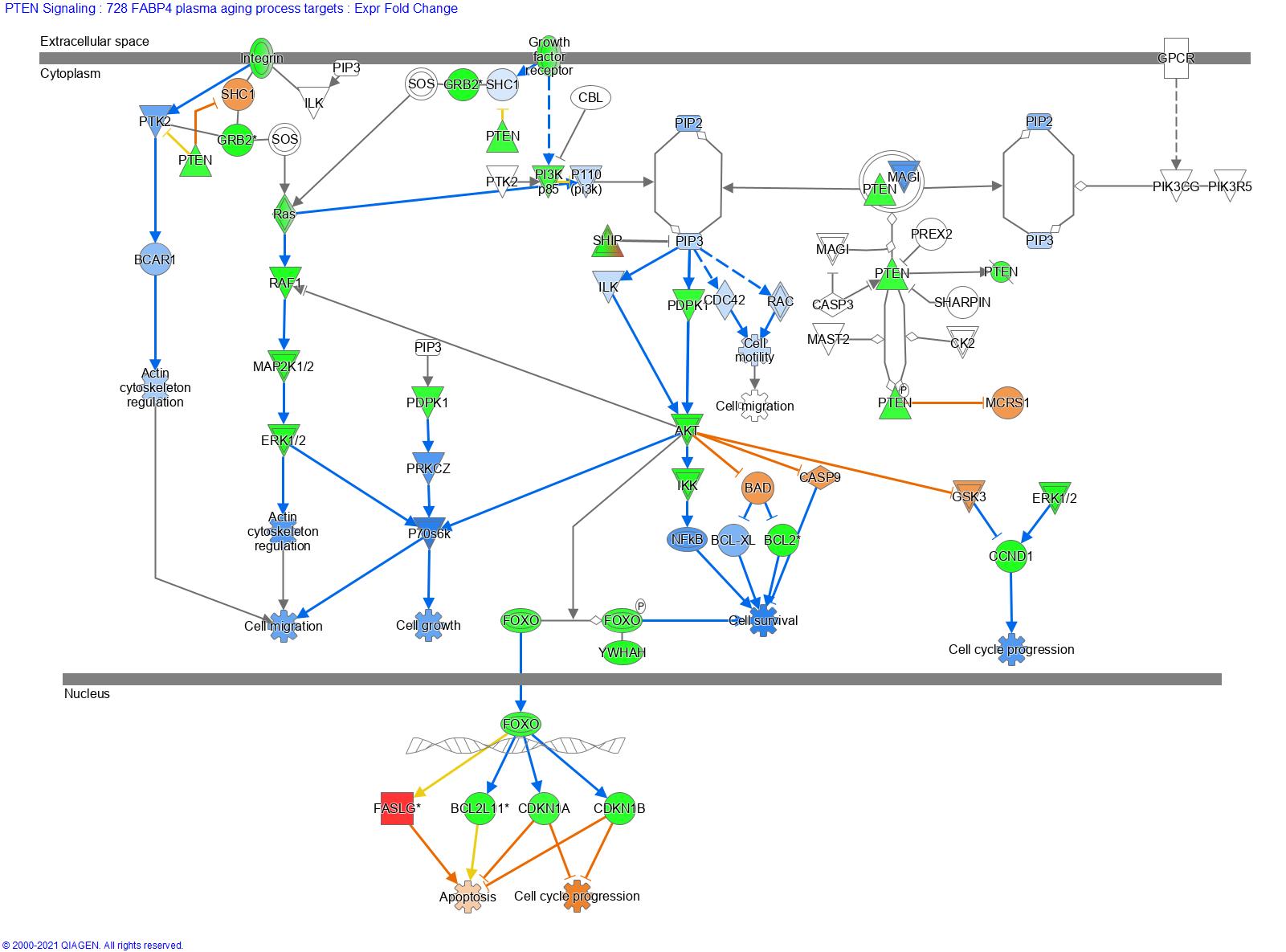


**S3 Figure 3.** Regulation of Cellular mechanisms by Calpain Protease


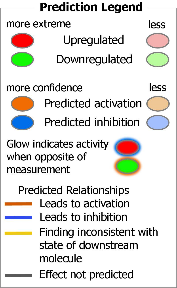

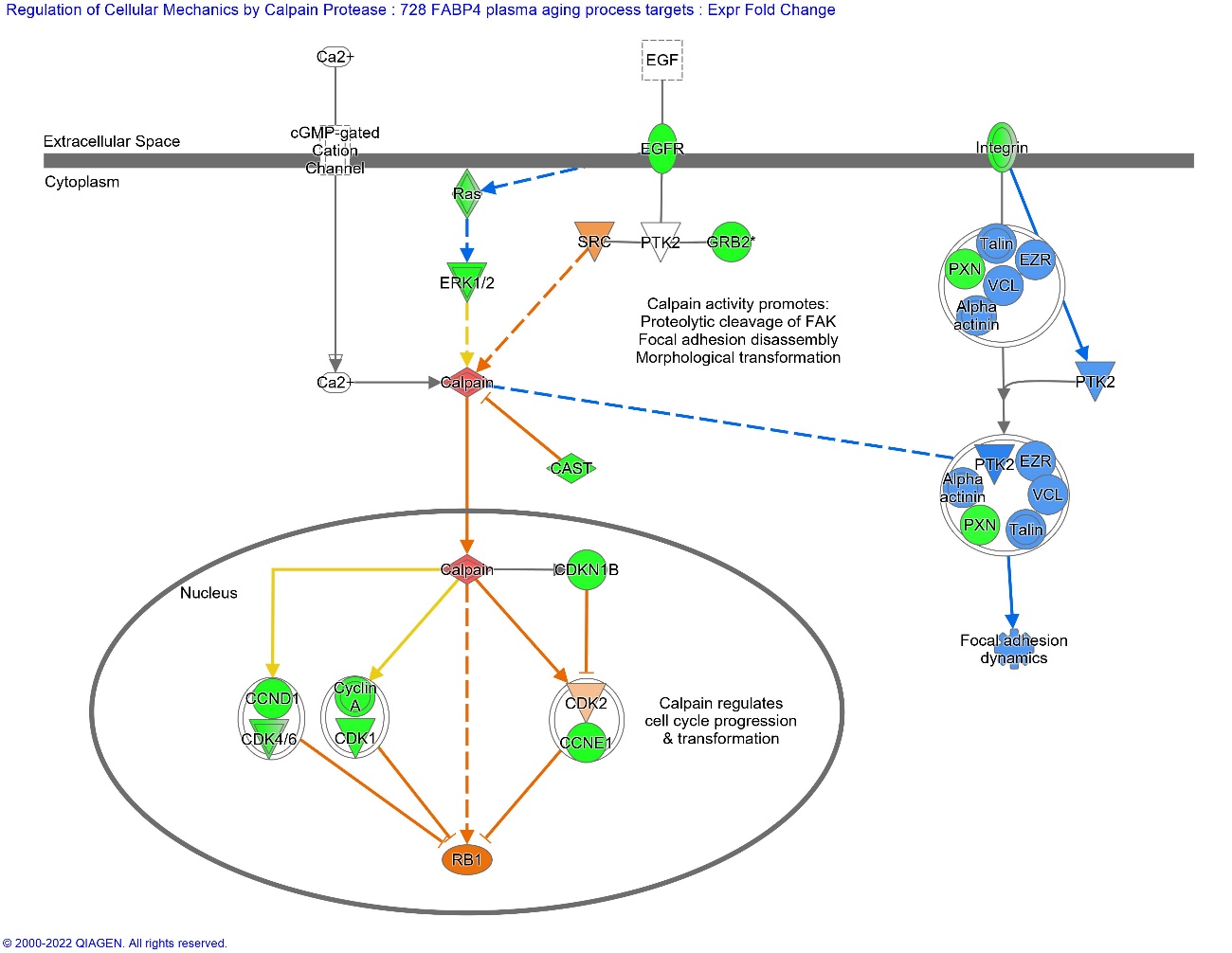


**S4 Figure 4.** Upstream Regulator as Network (miR-15b-5p)


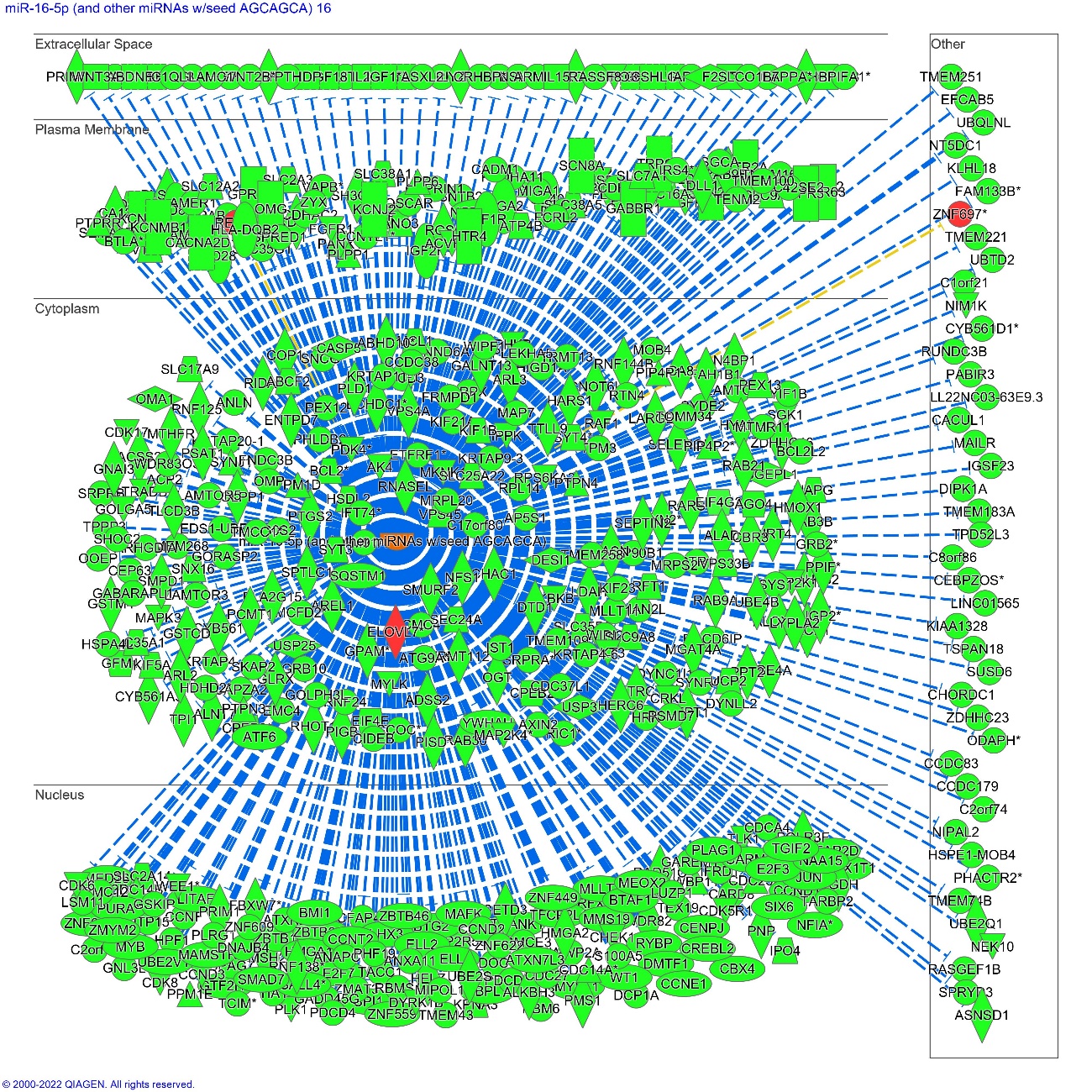

Supplement: Supplementary file 3 [file DataSheet1.docx]
